# *Haemophilus* Responses to Nutritional Immunity: Epigenetic and Morphological Contribution to Biofilm Architecture, Invasion, Persistence and Disease Severity

**DOI:** 10.1371/journal.ppat.1003709

**Published:** 2013-10-10

**Authors:** Blake R. Szelestey, Derek R. Heimlich, Forrest K. Raffel, Sheryl S. Justice, Kevin M. Mason

**Affiliations:** 1 The Research Institute at Nationwide Children's, Center for Microbial Pathogenesis, Columbus, Ohio, United States of America; 2 The Ohio State University College of Medicine, Department of Pediatrics, Columbus, Ohio, United States of America; Faculté de Médecine Paris Descartes, site Necker, France

## Abstract

In an effort to suppress microbial outgrowth, the host sequesters essential nutrients in a process termed nutritional immunity. However, inflammatory responses to bacterial insult can restore nutritional resources. Given that nutrient availability modulates virulence factor production and biofilm formation by other bacterial species, we hypothesized that fluctuations in heme-iron availability, particularly at privileged sites, would similarly influence *Haemophilus* biofilm formation and pathogenesis. Thus, we cultured *Haemophilus* through sequential heme-iron deplete and heme-iron replete media to determine the effect of transient depletion of internal stores of heme-iron on multiple pathogenic phenotypes. We observed that prior heme-iron restriction potentiates biofilm changes for at least 72 hours that include increased peak height and architectural complexity as compared to biofilms initiated from heme-iron replete bacteria, suggesting a mechanism for epigenetic responses that participate in the changes observed. Additionally, in a co-infection model for human otitis media, heme-iron restricted *Haemophilus*, although accounting for only 10% of the inoculum (90% heme-iron replete), represented up to 99% of the organisms recovered at 4 days. These data indicate that fluctuations in heme-iron availability promote a survival advantage during disease. Filamentation mediated by a SulA-related ortholog was required for optimal biofilm peak height and persistence during experimental otitis media. Moreover, severity of disease in response to heme-iron restricted *Haemophilus* was reduced as evidenced by lack of mucosal destruction, decreased erythema, hemorrhagic foci and vasodilatation. Transient restriction of heme-iron also promoted productive invasion events leading to the development of intracellular bacterial communities. Taken together, these data suggest that nutritional immunity, may, in fact, foster long-term phenotypic changes that better equip bacteria for survival at infectious sites.

## Author Summary

Clinical management of upper and lower respiratory tract diseases caused by nontypeable *Haemophilus influenzae* (NTHI) is a significant socioeconomic burden. Therapies targeting the pathogenic lifestyle of NTHI remain non-existent due to a lack of understanding of host microenvironmental cues and bacterial responses that dictate NTHI persistence. Iron availability influences bacterial virulence traits and biofilm formation; yet, host sequestration of iron serves to restrict bacterial growth. We predicted that fluctuations in availability of iron-containing compounds, typically associated with infection, would impact NTHI pathogenesis. We demonstrated that transient restriction of heme-iron triggered an epigenetic developmental program that enhanced NTHI biofilm architecture, directly influenced by induced morphological changes in bacterial length. Heme-iron restricted bacteria were primed for survival in the mammalian middle ear, due in part to an observed reduction in host inflammation coinciding with a striking reduction in host mucosal epithelial damage, compared to that observed in response to heme-iron replete NTHI. Moreover, transiently restricted NTHI were more invasive of epithelial cells resulting in formation of intracellular bacterial communities. Our findings significantly advance our understanding of how host immune pressure and nutrient availability influence pathogenic behaviors that impact disease severity.

## Introduction

Nontypeable *Haemophilus influenzae* (NTHI) is an obligate commensal that asymptomatically colonizes the human nasopharynx. Permissive risk factors, which include upper respiratory tract viral infection and Eustachian tube dysfunction, influence NTHI migration from the nasopharynx to sterile sites of the upper and lower respiratory tract [1], [2]. At these privileged sites, NTHI is a common causative agent of diseases such as otitis media (OM), conjunctivitis, sinusitis, pneumonia, and exacerbates disease severity in patients with chronic obstructive pulmonary disease and cystic fibrosis [3]–[8]. As observed with many other chronic, recurring infections, NTHI-mediated otitis media results from the development of robust organized biofilms [9]–[12]. However, the host and bacterial elements that dictate the complexity and fitness of NTHI biofilms are not completely understood.

As a mechanism to resist microbial outgrowth, the host tightly sequesters essential metals (i.e. iron, zinc, manganese) [13]. This process, termed nutritional immunity, results from the nearly universal requirement of specific host-derived nutrients for bacterial growth. One such molecule is the iron-containing metalloporphyrin, heme. Since NTHI lacks biosynthetic enzymes to generate protoporphyrin IX, the structural backbone of heme, import of an exogenous source of heme is necessary to support aerobic growth [14]. Low levels of heme-protein complexes can be released into serum upon erythrocyte lysis; however, free heme is quickly bound with albumin, hemopexin or haptoglobin molecules [15]. Heme sequestration ensures that the level of freely available iron within the host is far less than is required for microbial growth [16], [17]. Yet, NTHI utilizes multiple mechanisms to efficiently acquire heme from host heme sequestration complexes [18]–[21]. Intracellular heme is incorporated into cytochromes for respiration, catalase for enzymatic neutralization of oxygen radicals, and is essential for sequestration of free iron to minimize oxygen radical production [22].

Progression of NTHI disease is coincident with microenvironmental changes associated with migration to heme-iron restricted privileged sites. Studies investigating the effects of iron-restriction upon bacteria have demonstrated a range of physiological responses including increased antibiotic resistance, virulence factor expression as well as induction of biofilm formation [23]–[26]. We and others have demonstrated that NTHI upregulates iron uptake systems in the middle ear and that NTHI deficient in iron acquisition are less fit to survive in this environment [27]–[30]. Further, loss of Sap transporter function, an inner membrane ABC transporter critical for heme-iron homeostasis, results in clearance of NTHI from the chinchilla middle ear and nasopharynx [29], implicating a critical role for heme-iron acquisition in NTHI survival at these host sites. Moreover, loss of the SapF ATPase, required for energy dependent import of heme-iron, leads to the formation of biofilms with a lace-like architecture [31], suggesting a role for heme-iron availability in modulation of biofilm structure. These data indicate that lack of available essential nutrients (nutritional immunity) may significantly contribute to the NTHI pathogenic lifestyle. The rapid transcriptional responses of planktonic NTHI following restoration of heme-iron have been described [32]. However, the consequences of transient heme-iron restriction on biofilm development and influences on disease progression have yet to be determined.

We demonstrated that transient heme-iron restriction resulted in physiological changes that modified NTHI biofilm architecture, directly influenced by changes in bacterial morphology. These bacteria were primed for survival in the mammalian middle ear, due in part, to an observed reduction in host inflammation coinciding with a striking reduction in host mucosal epithelial damage, compared to the significant disruption in mucosal integrity observed in response to heme-iron replete NTHI. Our findings significantly advance our understanding of how host immune pressure and nutrient availability influence pathogenic behaviors that impact disease severity.

## Results

### Transient heme-iron restriction evokes NTHI community architectural changes

**Figure 1:**
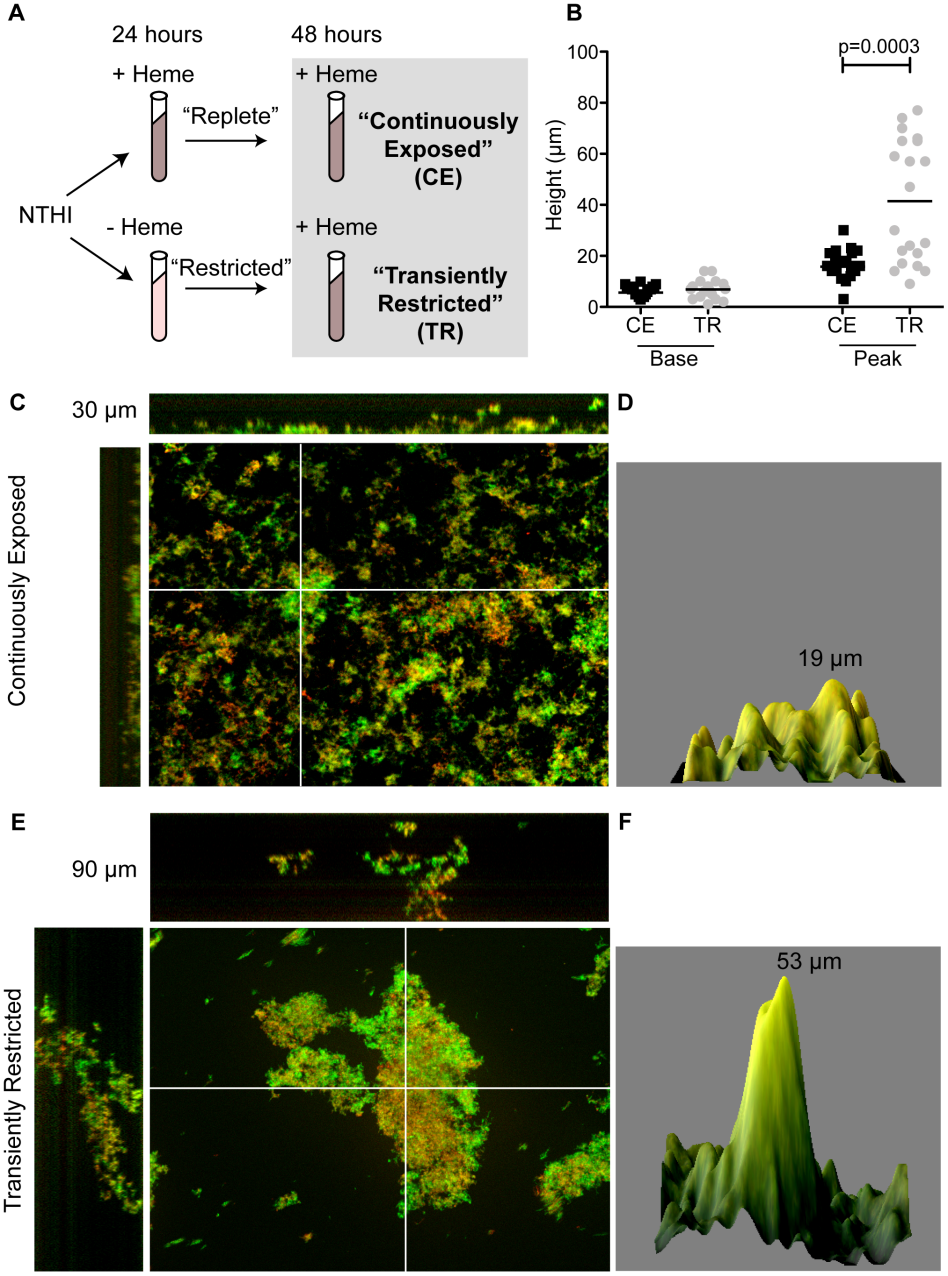
Transient restriction of heme-iron promotes biofilm tower architecture. Schematic representation of environmental heme-iron restriction. 86-028NP was cultured in the presence (+) or absence (−) of heme-iron for 24 hours generate “replete” and “restricted” populations, respectively. These populations were subcultured into medium containing heme-iron to produce continuously exposed (CE) or transiently restricted (TR) populations (**A**). The height of biofilm base or tower formed by CE or TR NTHI was measured in 20 random fields of view from a representative 48-hour biofilm (**B**). Statistical analysis was performed using a two-tailed t-test. Biofilms were visualized with live/dead stain, imaged using optical sections and reconstructed to generate a 3D view from the top (large pane) including orthogonal views of the side (top and left side) (**C, E**). Series of optical sections from an independent experiment were used to generate a surface plot to depict biofilm architecture (**D, F**). Images were processed using Image J and the height of the biofilm is indicated in each panel.

Scavenging and sequestration of heme-iron restricts the availability of this essential nutrient to bacteria. However, progression of disease coincides with increased inflammation and edema which, although also sequestered in host proteins, can provide a source of heme-iron to the bacterium. The consequence of these temporospatial fluctuations in heme-iron availability on NTHI virulence and persistence remains undetermined. We previously demonstrated that inactivation of the SapF ATPase, required for heme-iron import in NTHI, influenced biofilm architecture [31]. Whereas the use of engineered heme-iron deficient NTHI strains has contributed to the identification of a host microenvironmental cues that contribute to NTHI pathogenesis [31], [33], we sought to evaluate the effect of transient heme-iron restriction on wild type NTHI biofilm architecture and pathogenesis. To this end, we devised a culture-based system as a means to model temporospatial fluctuations of heme-iron availability during disease progression. Specifically, we cultured the prototypical NTHI strain, 86-028NP, in a defined medium in the absence or presence of heme-iron for 24 hours, to generate heme-iron ‘restricted’ and ‘replete’ individuals, respectively (Fig. 1A). Restricted or replete NTHI were subcultured into a medium containing 2 µg heme mL^−1^ to generate transiently restricted and continuously exposed NTHI, respectively. After 48 hours of growth, NTHI continuously exposed to heme-iron formed biofilms with peaks that reached heights of up to 30 µm (Fig. 1C). In contrast, we observed a difference in both frequency and height of peaks, extending up to 90 µm, following transient restriction of heme-iron (Fig. 1E, D, F; Fig. S1A, B). Notably, the differences in biofilm architecture were not due to changes in viability, adherence to an abiotic surface or growth rates following heme-iron restriction (Fig. S2A, B).

To better define the differences in the biofilms observed between the two experimental conditions, we quantitatively evaluated the architecture of the biofilms. The biofilms were comprised of two structurally distinct components, namely the biofilm base and the peaks. The biofilm base was defined as the distance from the abiotic surface to the lowest valley of the peaks in each of 20 randomly selected fields of view and represents the consistent coverage of biomass. The peaks were measured from the glass surface to the highest biofilm-associated signal observed in the field of view. There was no difference in average height (10 µm) of the base of the biofilms formed from transiently restricted or continuously exposed cultures (Fig. 1B). Peaks were observed with a range of 10–30 µm in overall height (Fig. 1B, C, D) in continuously exposed cultures. However, we observed that transient heme-iron restriction resulted in the formation of taller mushroom-shaped stalks up to 90 µm in height (Fig. 1B, E, F; p = 0.0003). This phenotype was maintained for at least 72 hours (Fig. S1C, D, E), suggesting that the transient restriction of heme-iron has long-standing physiological changes (i.e. transmission to daughter cells) that enhance the complexity and extent of biofilm development of NTHI. Thus, transient heme-iron restriction provokes developmental changes of biofilms grown on abiotic surfaces.

**Figure 2:**
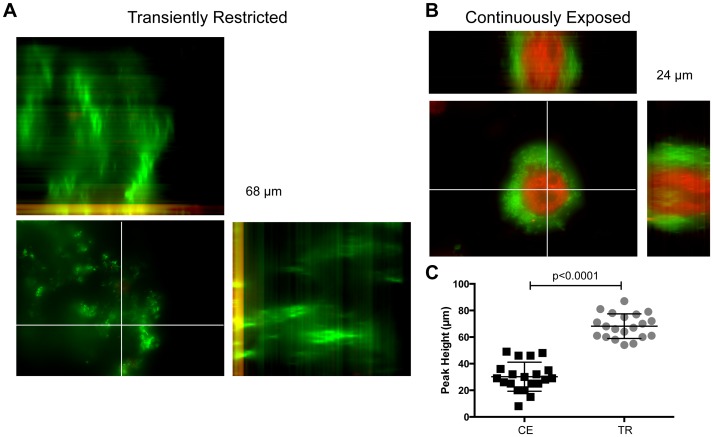
Transient restriction of heme-iron promotes biofilm tower architecture on epithelial cells. 86-028NP/pGM1.1 was cultured for 24 hours in the presence or absence of heme-iron to generate “replete” and “restricted” populations (CE or TR). Nutritionally conditioned NTHI were co-cultured with chinchilla middle ear epithelial cells for 48 hours. Epithelial cell surface was labeled with wheat germ agglutinin conjugated to Alexa-Fluor 594 (red). Biofilms were visualized by GFP fluorescence (green), imaged using optical sections and reconstructed to generate a 3D view from the top (large pane) including orthogonal views of the side (top and left side) (**A, B**). The height of biofilm tower formed by CE or TR NTHI was measured in 20 random fields of view from a representative 48-hour co-culture (**C**). Statistical analysis was performed using a two-tailed t-test.

We further investigated the propensity of heme-iron availability on biofilm architecture when grown on cultured chinchilla middle ear epithelial cells. We observed that transient heme-iron restriction resulted in the formation of biofilms with increased peak height (Fig. 2A) that range up to 84 µm in height as compared with a peak height up to 43 µm with heme-iron replete NTHI (Fig. 2B; p<0.0001). Therefore, the changes in biofilm architecture in response to heme-iron availability are observed on both abiotic and biotic surfaces.

### Community architectural height arises from transiently restricted NTHI in a mixed culture

**Figure 3:**
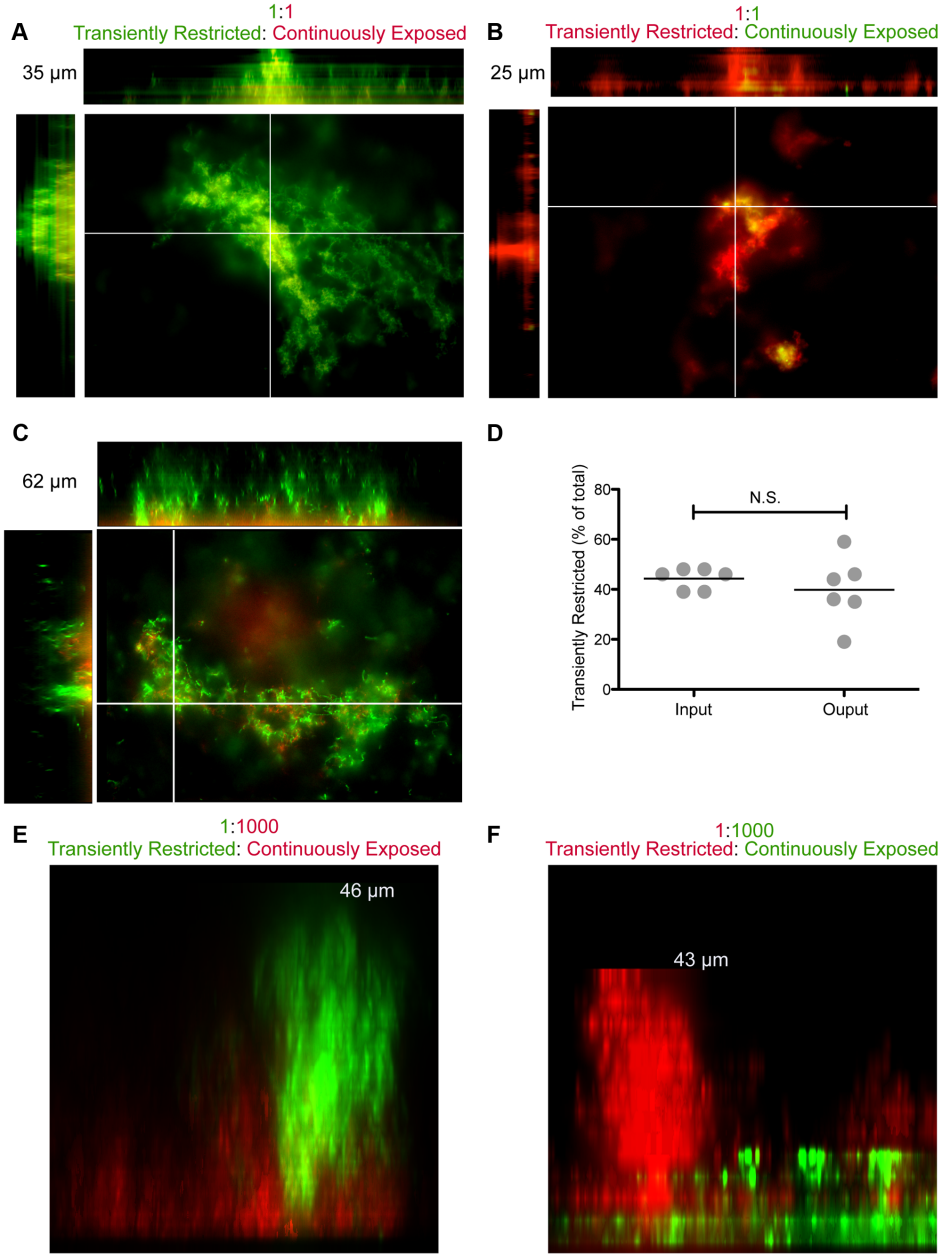
Robust towers arise from transiently restricted NTHI in a mixed culture. The reporter strains [86-028NP/pGM1.1 (production of GFP) and 86-028NP/pKM1.1 (production of mCherry)] were cultured for 24 hours in the presence or absence of heme-iron to generate “replete” and “restricted” populations. Restricted 86-028NP/pGM1.1 was co-cultured with equivalent numbers of replete 86-028NP/pKM1.1 for 48 hours in 2 µg heme mL^−1^ (**A**) or 20 µg heme mL^−1^ (**C**) in a glass chamber slide. Restricted 86-028NP/pKM1.1 was co-cultured with equivalent numbers of replete 86-028NP/pGM1.1 for 48 hours in 2 µg heme mL^−1^ (**B**) in a glass chamber slide. Optical sections were rendered to form the top down and orthogonal views. Biofilms formed from co-culture of restricted 86-028NP/pKM1.1 with replete 86-028NP/pGM1.1 were subjected to disruption to enumerate the total numbers of each reporter strain present following 48 hours of biofilm growth (**D**). Restricted 86-028NP/pGM1.1 was co-cultured with 1000 fold more 86-028NP/pKM1.1 replete for heme-iron for 48 hours in 2 µg heme mL^−1^ (**E**) in a glass chamber slide. Restricted 86-028NP/pKM1.1 was co-cultured with 1000 times more 86-028NP/pGM1.1 replete for heme-iron for 48 hours in 2 µg heme mL^−1^ (**F**) in a glass chamber slide. Orthogonal views of 3D rendered image are depicted (**E, F**). Heights of the biofilm are indicated in each panel. Statistical analysis was performed using a two-tailed t-test.

We next sought to assess the distribution of daughter cells within a biofilm formed from mixed cultures of transiently restricted and continuously exposed NTHI. In order to follow the lineage of each NTHI within the biofilm with regard to the heme-iron status at the time of biofilm initiation, we utilized two fluorescent reporter strains, one producing GFP and the other producing mCherry (both under control of the constitutively expressed outer membrane porin P2 promoter) [31]. Specifically, GFP-producing NTHI restricted of heme-iron for 24 hours were mixed in a 1∶1 ratio (based upon colony forming units; CFU) with mCherry-producing NTHI replete of heme-iron. Distribution of NTHI that arose from each culture condition was assessed following biofilm formation for 48 hours. We observed distinct segregation of the NTHI that originate from each culture condition. The biofilm peaks arose primarily from the transiently heme-iron restricted NTHI (Fig. 3A, C). The phenotype was independent of the fluorescent reporter used for transient restriction (Fig. 3B). Notably, equivalent numbers of each reporter strain were recovered at 48 hours of biofilm growth, indicating that the architectural differences observed were not due to changes in bacterial growth or viability (Fig. 3D). The biofilm base arose primarily from the NTHI that was continuously exposed to heme-iron (Fig. 3C). Given that the towers arose primarily from the transiently restricted NTHI, yet both reporter strains shared the same external environment, these data suggest that there is no transmission of phenotypes between NTHI of each origin. To further test this hypothesis, we co-cultured one transiently heme-iron restricted NTHI for every 1000 continuously heme-iron exposed NTHI (1∶1000) and assessed biofilm architecture and distribution of daughter cells. Again, we observed that the transient heme-iron restricted NTHI formed the towers of the biofilm regardless of which reporter strain experienced transient restriction, while the NTHI continuously exposed to heme-iron remained in the biofilm base (Fig. 3E, F). These data indicate that transient heme-iron restriction primes NTHI for enhanced biofilm formation that is transmitted to daughter cells during bacterial community development in a manner that is independent of growth rate, adherence to a surface or a soluble signal.

### Heme-iron restriction dictates community development and provides a competitive advantage during experimental otitis media

**Figure 4:**
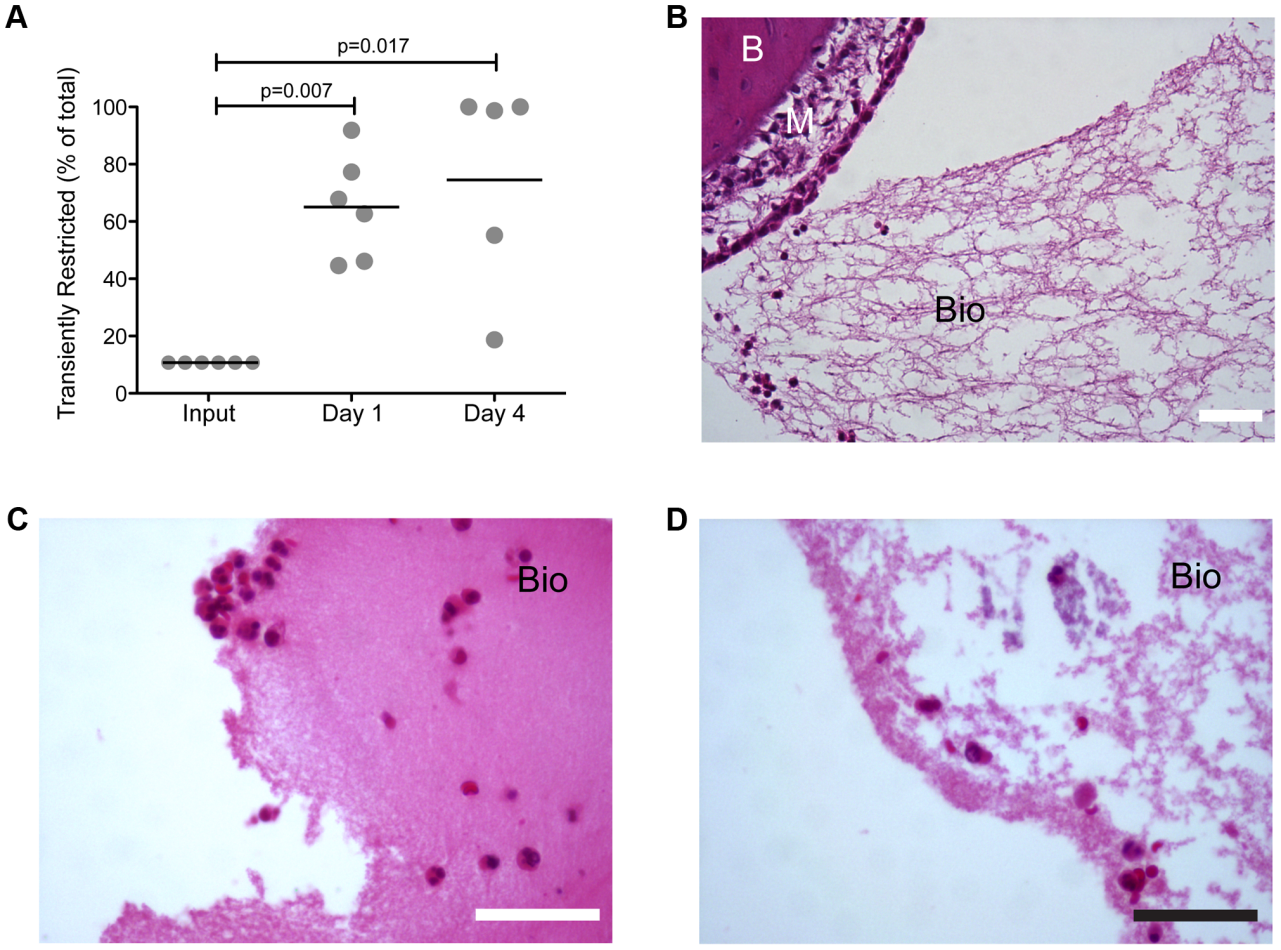
Heme-iron restriction provides a selective advantage during experimental otitis media in a mixed infection model. Restricted 86-028NP/pKM1.1 was mixed with 10 fold more 86-028NP/pGM1.1 replete for heme-iron for inoculation into the chinchilla middle ear. On days one and four post-inoculation, the total bacterial burden was enumerated from middle ear mucosal homogenates (**A**). The fluorescence emission spectrum was used to determine the origin of each colony. Statistical analysis was performed using a two-tailed t-test. Infected middle ear inferior bullae were analyzed by hematoxylin and eosin staining of thin sections with biofilm (Bio) as well as mucosa (M) and bone (B) highlighted (**B**). 86-028NP that was restricted (**D**) or replete (**C**) for heme-iron was individually inoculated into the chinchilla middle ears. Four days post inoculation, infected middle ear bullae were fixed and processed for histological examination. Scale bar = 50 µm.

We next sought to determine the ramifications of physiological changes associated with fluctuations in heme-iron availability on persistence and disease in a model of human otitis media. To this end, the heme-iron restricted mCherry reporter strain was mixed with the heme-iron replete GFP reporter strain (1∶10) and inoculated (total CFU = ∼2500 bacteria/ear) via transbullar injection into the chinchilla middle ear. Viable bacteria were enumerated one and four days post infection by homogenization of middle ear mucosa and the associated biofilm. Despite comprising only 10% of the inoculum, the originally heme-iron restricted population strongly out-competed the heme-iron replete population when enumerated from middle ear mucosa homogenates with a mean of 65 percent of NTHI recovered on day one and 75 percent on day four (with 3 of 5 ears over 90%)(Fig. 4A). This observation is in marked contrast to a similar experiment performed *in vitro* (Fig. 3D), whereby there was no competitive advantage for either population. Taken together, these observations indicate the originally restricted NTHI population has a significant survival advantage under host immune pressure.

The architecture of the bacterial biofilms formed in the middle ear during experimental otitis media was evaluated by hematoxylin and eosin staining of thin sections taken from fixed middle ears. We observed that the biofilms that fill the middle ear space are composed of bacteria and host immune cell infiltrate (Fig. 4B; Bio). We compared the biofilm architecture within middle ears that were previously infected with heme-iron restricted NTHI, heme-iron replete NTHI, or in combination (1∶10, respectively). We observed that prior heme-iron status resulted in changes in architecture during experimental OM. Within the middle ears that were infected with heme-iron restricted NTHI in combination with heme-iron replete NTHI, biofilms appeared to form an open lace-like architecture (Fig. 4B). Similar biofilm architecture was observed in the middle ears infected with only heme-iron restricted NTHI (Fig. 4D). In contrast, we observed condensed biofilm architecture within middle ears infected with heme-iron replete NTHI (Fig. 4C).

### Heme-iron availability influences morphological plasticity of NTHI

**Figure 5:**
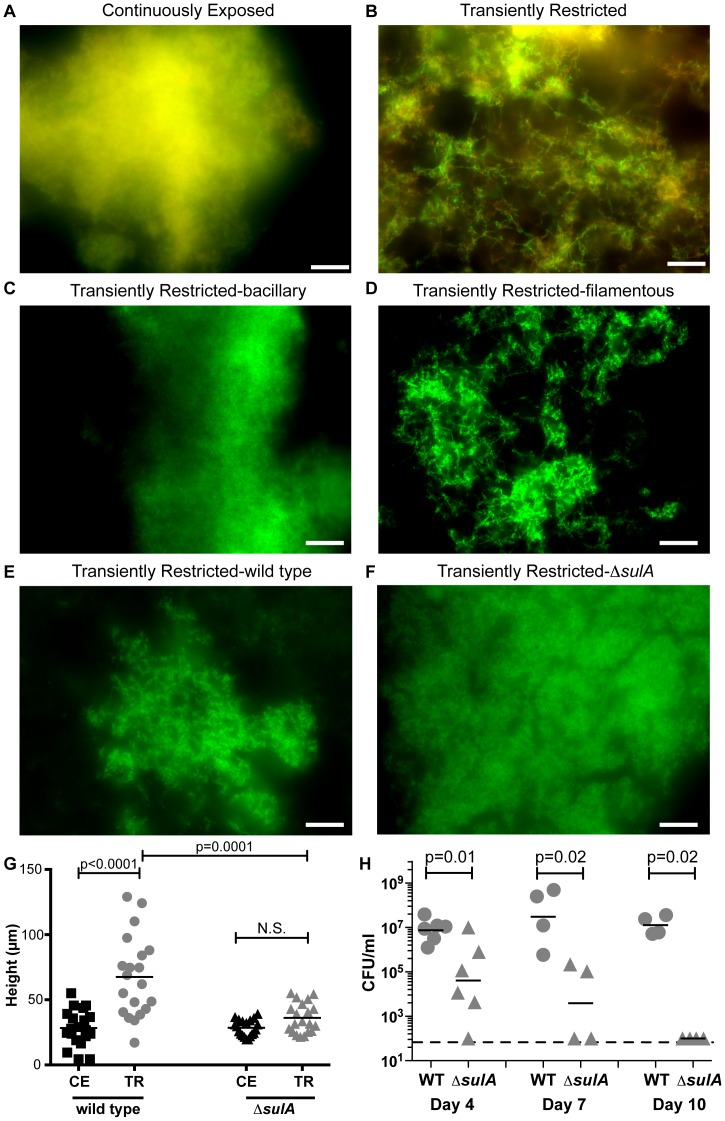
Lace-like architecture and towers arise from SulA-mediated filamentous morphotypes. Restricted or replete 86-028NP were cultured in medium containing 2 µg heme mL^−1^ for 48 hours on glass chamber slides and visualized by fluorescent microscopy using a live/dead stain (**A, B**). Restricted 86-028NP/pGM1.1 was grown in the presence 2 µg heme mL^−1^ for 6 hours and subjected to centrifugation through a 5 µm pore filter to enrich for bacillary and filamentous subpopulations. Enriched bacillary (**C**) and filamentous (**D**) populations were used to initiate biofilm formation on glass chamber slides in the presence of 2 µg heme mL^−1^ for 48 hours. Scale bar = 10 µm. Restricted 86-028NP/pGM1.1) (**E**) and restricted *sulA* (86-028NP Δ*sulA*/pGM1.1) (**F**) were used to initiate biofilm formation for 48 hours. The height of biofilm base or tower formed by CE or TR NTHI was measured in 20 random fields of view from a representative 48-hour biofilm (**G**). Statistical analysis was performed using a two-tailed t-test. 86-028NP/pGZRS39A and 86-028NP Δ*sulA*/pSPEC1 were restricted of heme-iron for 24 hours. Both strains were normalized for viability and equivalent numbers of each strain were co-inoculated into the middle ears of chinchillas. Effusions were collected on day 4, 7 and 10 days following inoculation and plated on selective media (**H**). Dashed line indicated limit of detection. Statistical analysis was performed using a Mann-Whitney U-test.

The observation that heme-iron restriction results in biofilms with a lace-like architecture in the middle ear (Fig. 4B), combined with our previous observations [31], suggested that these architectural changes may be related to heme-iron availability. To more closely examine the changes in biofilm infrastructure due to heme-iron restriction, we investigated individual bacterial interactions within the biofilm peaks *in vitro*. Microscopic evaluation at higher magnification revealed that biofilms derived from NTHI that were continuously exposed to heme-iron formed a dense, mat-like architecture, similar to that observed *in vivo* (compare Fig. 4C and Fig. 5A). In fact, individual bacteria are tightly associated and difficult to distinguish within the biomass. In marked contrast, the biofilm towers derived from transiently heme-iron restricted NTHI formed an open, lace-like architecture, similar to that observed *in vivo* (Compare Fig. 4D and 5B). The biofilm towers appeared to be constructed from strands of NTHI consisting of multinucleate filamentous morphotypes that interweave to form the lace-like architecture.

Filamentous NTHI have been observed on the middle ear mucosa of chinchillas with experimental OM [34]. In addition, filamentation in other bacterial species has been shown to occur in response to environmental stressors [35]. Taken together with our observation of the filamentous NTHI within the biofilms, we hypothesized that the transition from a heme-iron restricted to a heme-iron rich microenvironment would induce the filamentous morphotype observed. In order to determine the specific contribution of the filamentous populations to biofilm architecture, we evaluated the outcome of seeding for biofilm formation with cultures enriched for the filamentous or bacillary morphotype. The filamentous population was significantly enriched by retention on a 5 µm pore filter (Methods and Materials; [36]) while the bacillary population was obtained from the culture that passed through the filter. Biofilms that arose from the bacillary population were reminiscent of the biofilms composed of NTHI subjected to continuous exposure to heme-iron (Fig. 5C) forming a confluent, mat-like architecture. In contrast, the biofilms that arose from the population enriched for the filamentous morphotype displayed the open lace-like architecture that is characteristic of the transiently restricted population (Fig. 5D). Collectively, these data indicate that transient heme-iron restriction influences NTHI morphological changes in a subpopulation that contribute to biofilm architectural changes including the lace-like architecture and tower formation (Fig. S3).

### A SulA-related ortholog is required for NTHI filamentation and biofilm architecture associated with transient heme-iron restriction

**Figure 6:**
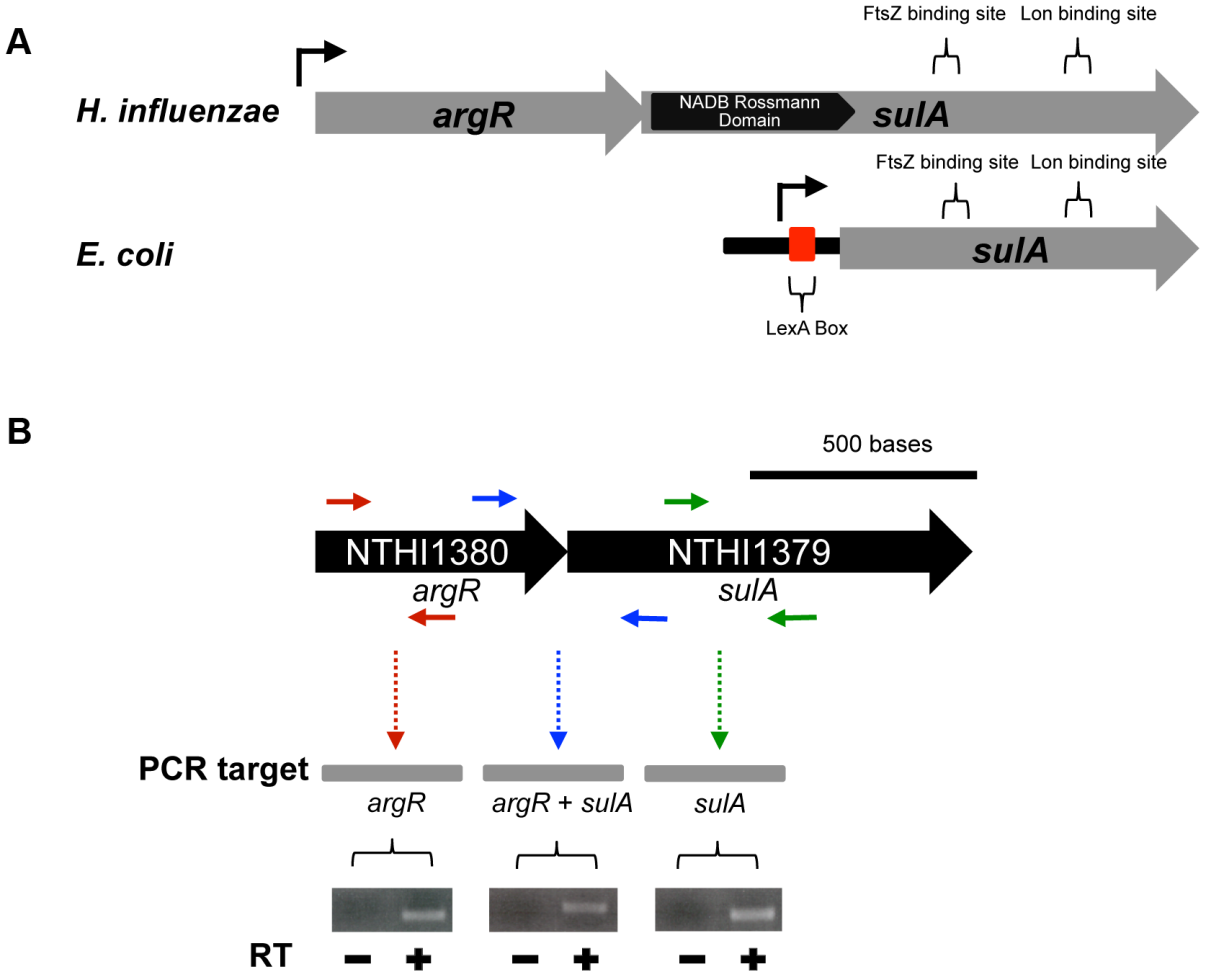
The NTHI SulA ortholog is encoded in a dicistronic operon. Schematic representation of the genetic context of the classical *sulA* (from *E. coli*) and the *sulA*-related ortholog in NTHI (**A**). See text for details. In contrast to the monocistronic message produced for the classical *sulA*, the SulA-related ortholog is produced from a dicistronic operon. RT-PCR was used to validate co-transcription of *argR* and *sulA* (**B**).

SulA is a component of the SOS response and is responsible for the inhibition of septation during repair of DNA damage [37]. This classically described SulA binds to the FtsZ monomer to prevent FtsZ polymerization at midcell resulting in the formation of non-septate filaments [38]–[40]. Septation is restored by Lon-mediated degradation of SulA, leading to polymerization of FtsZ at midcell [37]. We demonstrated that the NTHI *sulA* gene is part of an operon encoding the arginine repressor, *argR* (Fig. 6). This genetic context is unlike other SulA orthologues and is conserved amongst all sequenced *Haemophilus* strains. The NTHI SulA-related ortholog is almost twice as large as the classical SulA, yet retains sequence identity (70%) in the regions including the binding sites for FtsZ and the Lon protease, suggesting a functional role for NTHI SulA in bacterial filamentation.

We reasoned that this cell division inhibitor could be responsible for the filamentation observed following transient heme-iron restriction. To evaluate the contribution of filamentation to biofilm architecture, we constructed an unmarked deletion of *sulA* (Methods and Materials). Growth of the *sulA* mutant was indistinguishable from the wild type strain under laboratory conditions (Fig. S2C). In contrast to the lace-like architecture of the biofilms produced by the wild type strain (Fig. 5E), we observed a mat-like architecture of the biofilms formed following transient heme-iron restriction in the absence of SulA (Fig. 5F). In addition, there was a significant decrease in the overall peak height of biofilms in the absence of SulA (p = 0.0001)(Fig. 5G). Hence, the morphological changes and architecture of biofilms grown under transient heme-iron restriction required the activity of SulA, suggesting that filamentation is an important component of the architectural attributes associated with fluctuations in heme-iron availability.

Although the regulation of the *argR-sulA* transcript is not characterized, we have previously shown that the promoter for *argR* is upregulated during NTHI-mediated experimental otitis media indicating a potential role during pathogenesis [41]. This observation is consistent with evidence demonstrating a role for ArgR during pathogenesis in other bacterial species [42]–[44]. Previously it has been shown that *argR* expression, in NTHI strain 86-028NP, is not induced upon exposure to hydrogen peroxide [45]. These data suggest that regulation of *argR-sulA* in *Haemophilus* may be independent of the SOS DNA damage repair response, hallmarks of the classical *E. coli* SulA ortholog. To determine genes that are induced in other bacterial species as part of the SOS response (including *sulA*), other groups have used Mitomycin C (MMC) or other DNA damaging agents (e.g. UV light) [46]–[50]. To assess whether DNA damage induces expression of *argR-sulA* in NTHI, we exposed an NTHI GFP reporter strain (GFP under control of the *argR-sulA* promoter) to increasing concentrations of MMC and monitored fluorescence intensity as a measure of promoter activity. As previously published, MMC induces expression of the *E. coli sulA* gene in a dose dependent manner (Fig. S4A). In contrast, expression of NTHI *argR-sulA* in response to MMC is similar to fluorescence observed in the absence of promoter and absence of MMC (Fig. S4A). Consistent with these observations, we did not detect increased expression of *argR* or *sulA* in response to MMC exposure (Fig. S4B). Thus, these data suggest that the SulA ortholog is not a component of the SOS regulon and may represent a novel class of cell division inhibitors.

### The SulA-related ortholog is required for persistence during experimental otitis media

The contribution of SulA-mediated filamentation towards the persistence of NTHI was evaluated in an animal model of human otitis media. Chinchillas were co-infected with equivalent numbers of wild type NTHI and the *sulA* mutant. We observed significant loss of the *sulA* mutant in middle ear effusions as early as 4 days following infection (Fig. 5H). Although the wild type strain continued to persist at high levels (approx. 10^7^cells/ml effusion) at 10 days following infection, all effusions were devoid of detectable *sulA* mutant NTHI (Fig. 5H). Moreover, the *sulA* mutant was not recovered from disrupted epithelial mucosa at 12 days post infection. These data demonstrate that SulA contributes to the persistence of NTHI during pathogenesis.

### Disease severity is reduced in response to infection with heme-iron restricted NTHI

**Figure 7:**
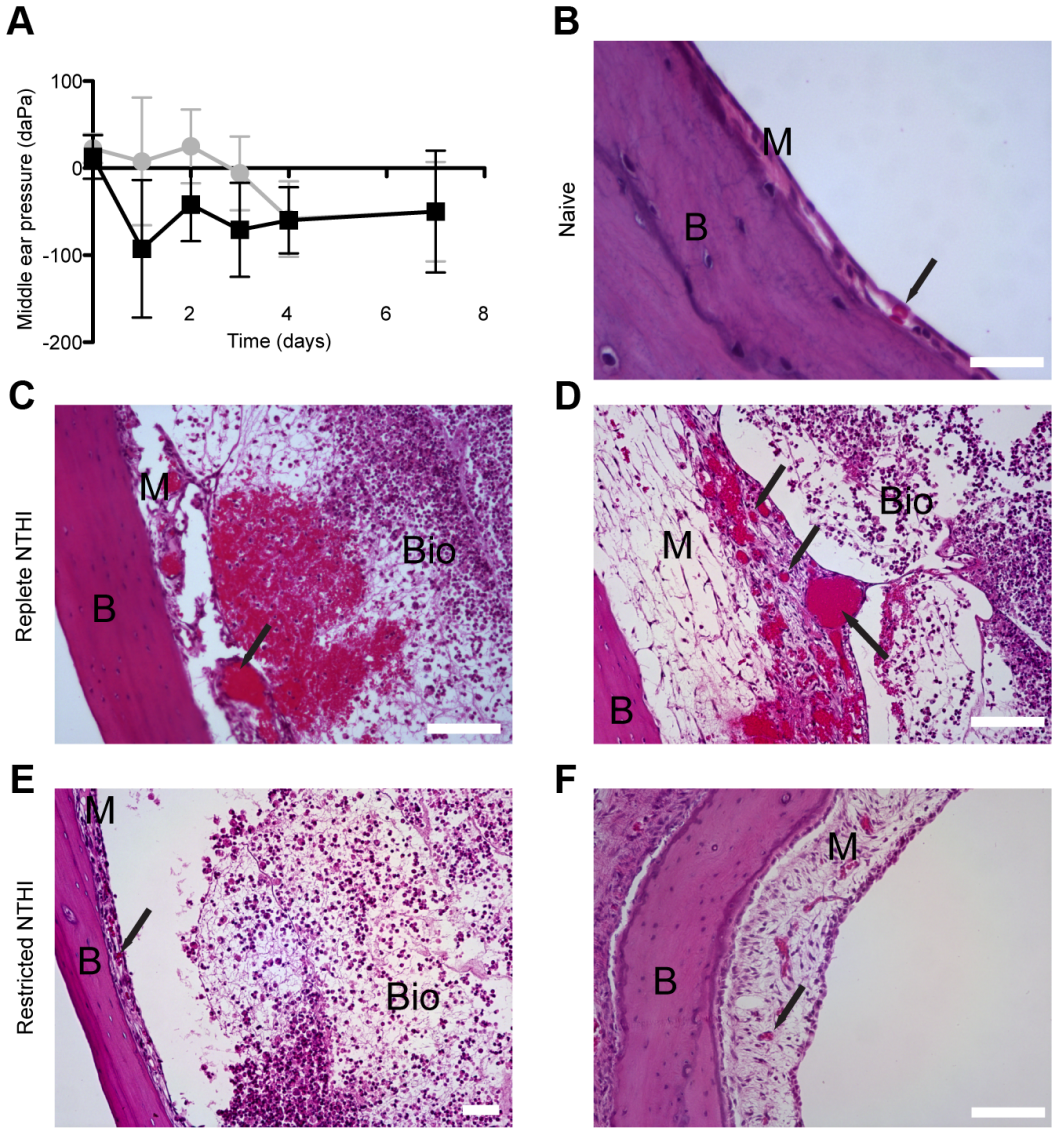
Heme-iron status influences disease severity of NTHI. Middle ear pressure was measured by tympanometry (mean ± S.D.) on each day following transbullar introduction of heme-iron replete (black squares) or restricted (gray circles) NTHI (**A**). Histopathology was assessed by examination of hematoxylin and eosin stained thin sections of naïve (**B**) or infected middle ear bullae 7 days following introduction of heme-iron replete (**C, D**) or restricted (**E, F**) NTHI. Scale bar = 25 µm (**B**), 50 µm (**F**), 100 µm (**C, D, E**). Abbreviations = Bone (B), mucosa (M), biofilm (Bio). Arrows denote capillaries.

We demonstrated that heme-iron restriction imparts a survival advantage for NTHI *in vivo* (Fig. 4A). Moreover, we observed that a change in bacterial morphology and subsequent influence on NTHI biofilm architecture and persistence during disease (Fig. 5) suggests that prior heme-iron status may also contribute to disease severity. Thus, we sought to characterize changes in disease progression following middle ear infection with NTHI, either restricted or replete for heme-iron at the time of infection. We first monitored middle ear pressure by tympanometry over the first seven-days following introduction of NTHI. A change in middle ear pressure is a clinical hallmark of otitis media [51]. During the acute phase of the infection and prior to any detectable middle ear effusion (day 1), we observed a rapid decrease in middle ear pressure that was detected in the cohort that received heme-iron replete NTHI (Fig. 7A). In contrast, we observed a delayed decrease in middle ear pressure in the cohort infected with the heme-iron restricted NTHI (day 2–4). These data are indicative of a change in the kinetics of disease progression based upon the prior heme-iron availability to NTHI. Notably, the difference in kinetics was not due to differences in bacterial burden per gram of tissue under these experimental conditions on day 7 [restricted NTHI 5.2×10^7^CFU/g; replete NTHI 3.8×10^8^ CFU/g: N = 6: p = 0.285].

To better characterize changes in bacterial-host interactions between the two cohorts, middle ear inferior bullae were examined histologically seven days following infection with either heme-iron replete or heme-iron restricted NTHI. We observed severe edema, vasodilatation, erythema, hemorrhage and host immune cell infiltrate with evidence of mucosal epithelial destruction in middle ears challenged with heme-iron replete NTHI (Fig. 7C, D), hallmarks of middle ear disease not observed in the naïve middle ear (Fig. 7B). In stark contrast, middle ears infected with heme-iron restricted NTHI were edematous with host immune cell influx; however, there was a clear absence of other hallmarks of disease severity (Fig. 7E, F). In fact, we observed an intact mucosal epithelial barrier to the lumen of the middle ear (Fig. 7E, F). These observations, concurrent with the changes in middle ear pressures observed between the two cohorts, support differential disease severity, dependent upon prior heme-iron status of NTHI. Collectively, our observations indicate that transient restriction of heme-iron influences NTHI morphology and biofilm architecture that imparts a survival advantage in the middle ear. Examination of bacteria-host interaction in the middle ear suggests that this survival advantage may be due, in part, to a differential host response, one of decreased disease severity, as a consequence of the prior heme-iron status of NTHI.

### Heme-iron restriction promotes internalization of NTHI leading to the formation of intracellular bacterial communities

**Figure 8:**
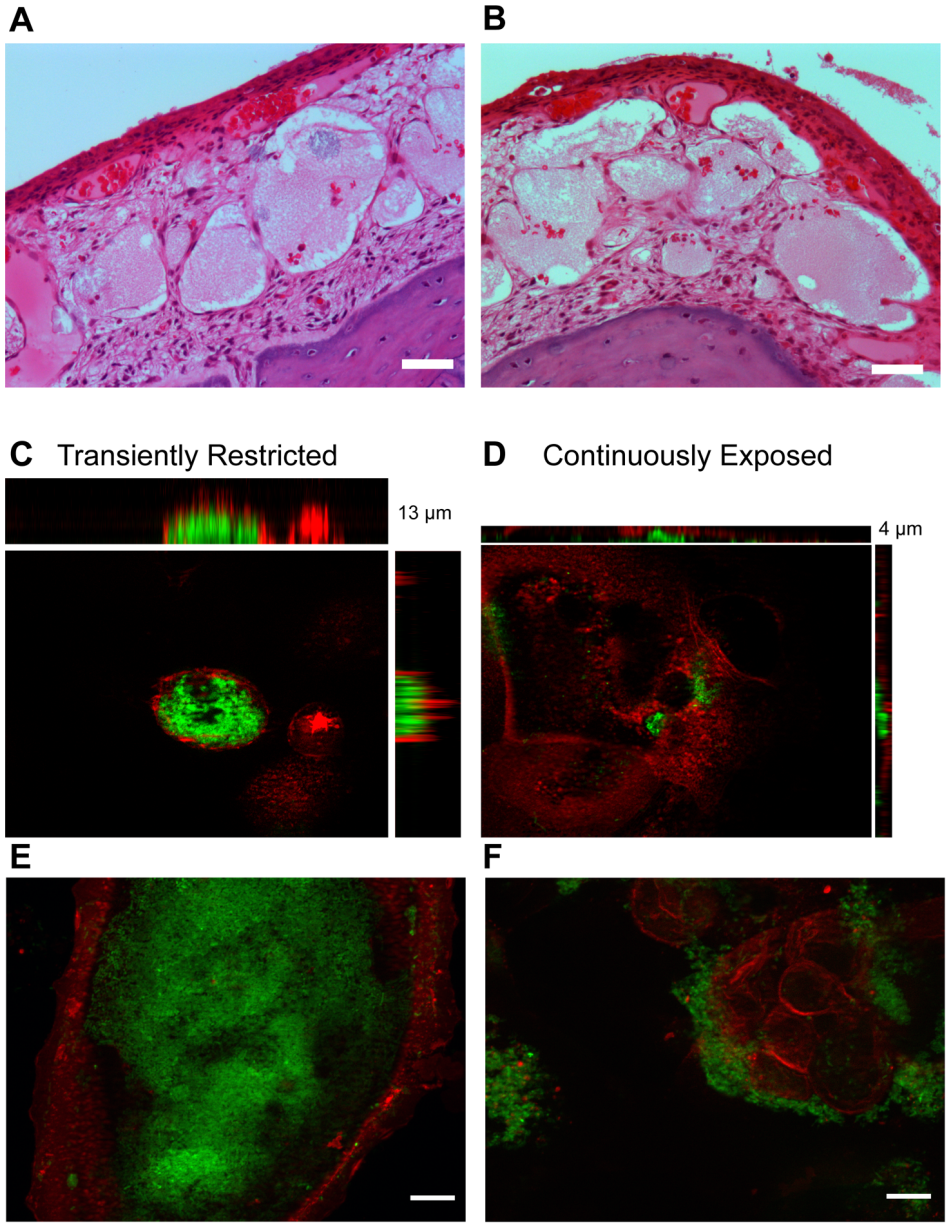
Transient restriction promotes intracellular bacterial community formation. 86-028NP that was restricted (**A, B**) for heme-iron was individually inoculated into the chinchilla middle ears. Four days post inoculation, infected middle ear bullae was fixed and processed for histological examination. Scale bar = 10 µm. 86-028NP/pGM1.1 was cultured for 24 hours in the presence or absence of heme-iron to generate “replete” and “restricted” populations (CE or TR). Nutritionally conditioned NTHI were co-cultured with chinchilla middle ear epithelial cells for 48 hours. Epithelial cell surface was labeled with wheat germ agglutinin conjugated to Alexa-Fluor 594 (red). Biofilms were visualized by GFP fluorescence (green), imaged using optical sections and reconstructed to generate a 3D view from the top (large pane) including orthogonal views of the side (top and left side) (**C, D**). Optical sections that depict additional representative images of the localization of NTHI based upon nutritional conditioning (**E, F**). Scale bar = 10 µm.

We have previously demonstrated that NTHI defective in heme acquisition due to a mutation in the Sap transporter were hyperinvasive and were observed in the cytoplasm of epithelial cells [33]. In contrast, the wild type strain primarily colonized the apical surface of the epithelial cells and demonstrated reduced invasion events which were associated with vacuolar localization and NTHI clearance [33]. During our microscopic evaluation of mucosal tissue integrity as a consequence of prior heme-iron status, we observed what appear to be populations of NTHI within the epithelium of the chinchilla middle ear (Fig. 8A, B). NTHI populations were not readily observed in the epithelium of middle ears infected with heme-iron replete NTHI (Fig. 7). To confirm that the prior heme-iron status contributes to the invasion of NTHI, we investigated the frequency of invasion using our cultured epithelial cell model of infection. Following 48 hours of infection with heme-iron restricted NTHI, we observed numerous intracellular bacterial populations that appear to fill the volume of the cell (Fig. 8C, E). In contrast, when epithelial cells were infected with heme-iron replete NTHI, we observed fewer infected cells and those infected cells contained fewer bacteria (Fig. 8D, F). The ability of NTHI to fill the volume of the cell following restriction of heme-iron is suggestive that nutritional conditioning allows NTHI to escape vacuolar trafficking to gain access to the cytoplasm. This intracellular localization provides an additional compartment for bacterial growth and protection from host immune clearance mechanisms. Moreover, intracellular lifestyles are commonly associated with chronic and recurrent infections. Our experimental evidence demonstrated the establishment of a productive intracellular population as a consequence of enhanced invasion rates and fates due to nutrient limitation of wild type NTHI.

## Discussion

**Figure 9:**
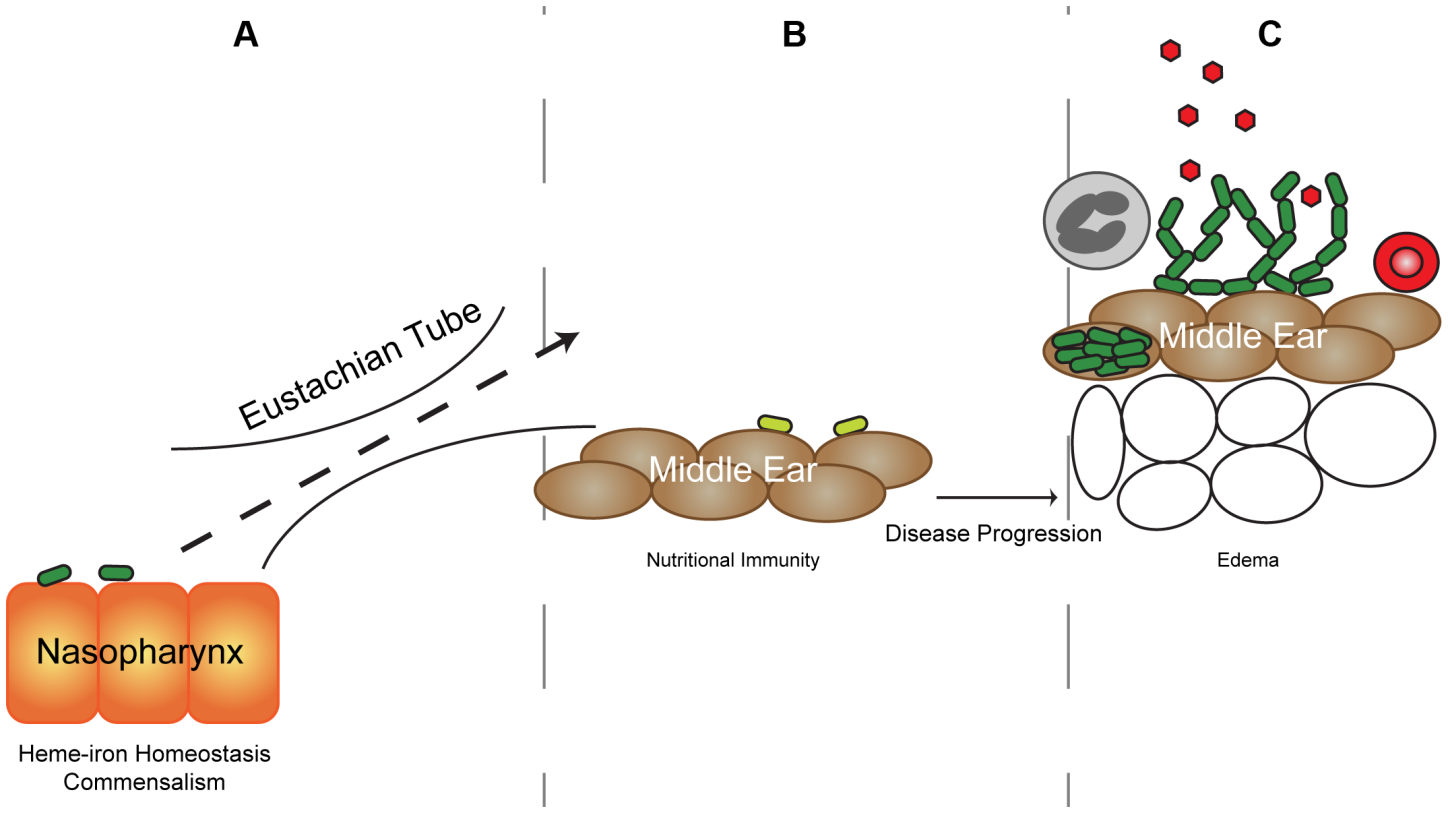
Proposed model for host-mediated nutritional environment effects on NTHI pathogenesis. NTHI (dark green bacteria) asymptomatically colonizes the heme-iron sufficient nasopharynx epithelium (orange cells) (**A**). Permissive factors promote ascension up the Eustachian tube to the intact middle ear epithelium (brown cells) where NTHI are heme-iron restricted (light green bacteria) (**B**). Disease progression results in infiltration of red blood cells (red circle), immune cells (gray circle) and expansion of interstitial space (open circles) leading to potential heme-iron sources (red hexagon) for NTHI (dark green bacteria). This transition from heme-iron restricted to replete microenvironments primes NTHI to alter morphology and enhance biofilm formation, both of which promote invasion and persistence (**C**).

The phenotypic changes associated with transient heme-iron restriction invoke an interesting paradox in which host nutritional immunity, which serves to scavenge essential nutrients [52] and thus prevent bacterial growth, conversely enhances persistence and reduces disease severity. Transition to the middle ear exposes NTHI to an iron-restricted environment, yet progression of disease coincides with a potential increase in availability of iron sources due to host inflammatory responses (Fig. 9). Given the near global bacterial requirement for iron, there have been many investigations into the mechanisms required for bacterial survival in iron-restricted microenvironments [53]–[55]. Interestingly, we observed that prior heme-iron restriction had dramatic impact on NTHI persistence in the middle ear as evidenced by enhanced survival in direct competition with heme-iron replete NTHI in an animal model for human otitis media (Fig. 4). The bacterial burden was indistinguishable when middle ears were infected with only heme-iron replete or heme-iron restricted NTHI. However, disease severity was attenuated in response to infection with heme-iron restricted NTHI (Fig. 7). We observed a lack of middle ear pathology (i.e. vasodilatation, hemorrhage, erythema, loss of mucosal epithelial integrity) and unremarkable tympanometry during infection with heme-iron restricted NTHI. Moreover, heme-iron restricted NTHI were more invasive of host epithelium, forming communities of intracellular bacteria within the epithelial cell cytoplasm (Fig. 8). Taken together, our data indicate that prior heme-iron restriction influences biofilm architecture, modulates the host immune response, and provides multiple mechanisms to persist in the face of the proinflammatory response.

Distinct from acute otitis media associated with middle ear inflammation, fever and pain, studies have identified an asymptomatic subclinical variant of otitis media that is primarily associated with middle ear effusion in the absence of appreciable tympanic pathology [56], [57]. This variant of otitis media with effusion can lead to chronic otitis media associated with bacterial persistence. We interpret the clinical parameters and histological data obtained from chinchilla middle ears infected with heme-restricted NTHI to closely mimic this asymptomatic variant of otitis media, and thus serve as a useful model to study this clinically relevant variant of middle ear disease. Moreover, the experimental conditions explored within provide tools to define the ramifications of host-pathogen interactions on disease progression and severity.

Although classically considered an extracellular, opportunistic pathogen, there is increasing evidence of *intra*cellular and *para*cellular niches for NTHI *in vitro* [58]. Invasion into epithelial cells could provide NTHI with an environment rich in nutrients and a refuge from immune pressures and thus provide a temporary or long-term respite from nutrient limitation and innate immune components. It is plausible that bacterial responses to fluctuations in nutrient availability modulate host pathogen interactions with the epithelium. In fact, NTHI that exhibit heme-iron restriction due to genetic manipulation (i.e. loss of Sap-mediated heme-iron transport) are hyper-invasive in a polarized epithelial model of infection [33]. Moreover, this hyperinvasion coincides with a change in intracellular trafficking that promotes *Haemophilus* survival in the cytoplasm [33]. We now demonstrate that heme-iron restriction influences NTHI invasion resulting in the formation of infected cells or pods that contain intracellular bacterial communities [59]–[61]. In addition to the potential for intracellular populations within the middle ear, the presence of NTHI within adenoids and bronchial epithelium suggests that an invasive phenotype may coincide with the chronic nature associated with NTHI-mediated diseases [62]–[66]. The chronic nature of NTHI infections including recalcitrance to antibiotic therapy, persistence in the presence of bactericidal antibodies and culture-negative clinical analysis are suggestive of biofilm formation and development of intracellular bacterial reservoirs within host cells [58], [62]–[64], [66]–[70]. Invasion and subsequent emergence of a viable reservoir of NTHI from the *intra*cellular and *para*cellular niches could serve as a seed for recurrent and chronic OM. Future studies will determine whether intracellular pools of *Haemophilus* provide a reservoir for persistence and recurrent infection.

The biological complexity of NTHI lifestyles contributes to pathogenesis and, importantly, to the prolonged, recurrent and difficult-to-treat nature of NTHI-induced disease. Co-culture of transiently restricted and replete NTHI during biofilm formation demonstrated that the spatial distribution of NTHI is a consequence of prior heme-iron status, which provides insight into the mechanisms that dictate biofilm architecture. We demonstrated that these changes are only transmitted from mother to daughter cells and are not transmitted to other bacteria within the same culture. This suggests that the observed changes in community structure are not likely mediated by a released soluble factor as all bacteria are exposed to the same microenvironment, yet only a subset proceed through a unique developmental program. Moreover, we observed a phenotypic change that persisted for at least 72 hours both *in vitro* and *in vivo*. The long-standing phenotypic changes observed both *in vivo* and *in vitro* following transient heme-iron restriction suggest that epigenetic changes accompany the fluctuations in nutritional availability. To begin to evaluate these long-term changes, the protein profiles of NTHI transiently restricted of heme-iron were compared with NTHI continuously exposed of heme-iron. Proteins known to be involved in heme-iron acquisition were present at similar levels, indicating that the transiently restricted NTHI have returned to heme-iron homeostasis within 6 hours following restoration of heme-iron (Table S1). We did observe increased levels of proteins involved in metabolism and oxidation-reduction (Table S1), suggesting that these changes could contribute to the enhanced persistence phenotype observed during otitis media (Fig. 4). Iron starvation triggers a DNA methylase-dependent epigenetic program to control gene expression in *Escherichia coli* [71]. We are currently investigating the potential role of DNA methylation in the epigenetic programs that promote distinctly different NTHI phenotypes that subsequently modulates disease manifestation.

Subpopulations have been observed in response to iron availability during biofilm development of *E. coli* [72]. We also observed that transient heme-iron restriction resulted in two phenotypically distinct populations that could be separated based upon the bacterial morphology (Fig. 5). We further demonstrated that the SulA-related ortholog was necessary to support the morphological plasticity, biofilm architectural changes and persistence associated with transient heme-iron restriction. We and others have demonstrated that morphological changes promote survival in the face of antimicrobial agents as well as the killing by professional phagocytes and predation [35], [36], [73], [74]. We propose that the morphological changes observed with NTHI would provide similar mechanisms for survival within the middle ear.

The observation that *sulA* is co-transcribed with *argR*, and that the genetic context is conserved in *Haemophilus* strains, suggests a previously undescribed regulatory mechanism for expression. We have shown that *argR-sulA* is not a constituent of the SOS regulon. Our data indicate that transient heme-iron restriction leads to NTHI filamentation, and that this morphological change is SulA-dependent. These observations raise the question whether *sulA-argR* regulation is heme-iron dependent, whether direct or indirect. We have shown that neither *argR* nor *sulA* genes are regulated by the iron-responsive regulator Fur [28], indicating a Fur-independent mechanism of iron regulation. In addition the role of arginine metabolism in *Haemophilus* persistence is unknown and under investigation.

Our observations highlight the impact that immune pressure and host microenvironments have on bacterial lifestyles. Here, we demonstrated that these changes in the developmental program equip NTHI to more successfully persist during disease progression. The nutrient limitation we imposed *in vitro* mimics nutritional immunity in the host and can be exploited to further elucidate the molecular details of bacterial developmental programs in response to host pressures during disease progression.

## Materials and Methods

### Ethics statement

All animal experiments were carried out in strict accordance with the accredited conditions in the Guide for the Care and Use of Laboratory Animals of the National Institutes of Health. The protocol was approved by the Institutional Animal Care and Use Committee (Welfare Assurance Number A3544-01) at The Research Institute at Nationwide Children's, AR13-00026. All experimental procedures were performed under xylazine and ketamine anesthesia, and all efforts were made to minimize suffering.

### Bacterial strains

NTHI strain 86-028NP is a minimally passaged clinical isolate which has been extensively characterized in the chinchilla model of human otitis media, and sequenced [45], [75]. Construction of a nonpolar unmarked deletion of the *sulA* gene was performed by the recombineering strategy as previously described [31], [76]. Briefly, primers 5′-CAGCCGCAATCGCAACAGTAGTAT-3′ and 5′-TTGGGTAAACGTGAAGAAAATG-3′ were used to amplify *sulA* and 1 kb of the flanking DNA both 5′ and 3′ to *sulA*. The subsequent amplicon was ligated into the pGEM-T Easy vector (pDH001) and transformed into *E. coli* strain DY380. In parallel, primers, 5′-GCTGAATTATTGCAAAATATCCAACGTTTATTTGAAAACGCATTGTAATGATTCCGGGGATCCGTCGACC-3′and 5′-TCACCGCATTAAAAAAGTGCGGTGATTTTTTATTTGTTTTTTAGGATTTCTGTAGGCTGGAGCTGCTTCG-3′, each containing 50 bp of DNA homologous to the 5′ and 3′ ends of the *sulA* gene, were used to amplify the spectinomycin cassette from pRSM2832 [76]. This amplicon was then electroporated into strain *E. coli* DY380/pDH001 to form strain DY380/pDH002, in which the *sulA* gene in pDH001 was been replaced by the spectinomycin cassette. The plasmid pDH002 was then used to transform NTHI 86-028NP, and transformants were selected by growth on spectinomycin-containing chocolate II agar plates. To generate a nonpolar deletion mutant, the *sulA* mutant was transformed with plasmid pRSM2947 and grown at 32°C, and FLP expression was induced using anhydrotetracycline. The cells were cured of the plasmid by growth at 37°C. The mutant strains were screened for growth on chocolate agar II (Becton Dickinson, Sparks, MD) and in parallel, susceptibility to spectinomycin, generating strain NTHI 86-028NP::*sulA* and were confirmed by sequence analysis.

Green fluorescent protein (GFP) reporter parent and *sulA* mutant strains were created by electroporation of pGM1.1 as published previously [31]. In order to generate an mCherry reporter strain, plasmid (pGM1.1) was isolated from 86-028NP/pGM1.1 and digested with *Bam*H1/*Eco*R1 to remove the *gfp* open reading frame. The open reading frame encoding mCherry was isolated as a *Bam*H1/*Eco*R1 restriction fragment from pRSET-B (Gift of Dr. Roger Tsien) [77] cloned into the *Bam*H1/*Eco*R1 site of pGM1.1 generating plasmid pKM1.1. The plasmid was used to transform NTHI 86-028NP, generating strain NTHI 86-028NP/pKM1.1.

To distinguish strains in a competition model of otitis media, 86-028NP and 86-028NPΔ*sulA* were marked as previously described [28]. 86-028NP was transformed with pGZRS-39A, a *Haemophilus-Actinobacillus pleuropneumoniae* shuttle vector that contains the kanamycin resistance gene from Tn*903* [78]. 86-028NPΔ*sulA* was transformed with pSPEC1, a variant of pGZRS-39A, in which the kanamycin resistance gene was replaced by a spectinomycin resistance gene [30].

### Environmental heme restriction

NTHI was grown overnight on chocolate II agar (Becton Dickinson, Sparks, MD) at 37°C in 5% CO_2_. Individual colonies were adjusted to an OD_490_ of 0.65 into chelated defined iron-source medium (DIS), diluted 10-fold and then subcultured into nitric acid-washed 15 ml round bottom glass tubes containing pre-warmed DIS medium with either 0, or 2 µg heme mL^−1^, and grown statically for 24 hours to deplete or maintain internal stores of heme, as previously described [18], [31]. Following depletion of heme-iron, bacteria were subsequently inoculated into DIS supplemented with 0, 2, or 20 µg heme mL^−1^ (Sigma Aldrich, St. Louis, MO) and statically grown at 37°C in 5% CO_2_ as previously described [18], [31]. Following incubation, the cultures were equilibrated to an OD_490_ of 0.37 and diluted appropriately into DIS containing 2 or 20 µg heme mL^−1^ to match cultures to 1×10^7^ CFU/ml as described for each experiment.

### Biofilm growth on an abiotic surface

Cultures (50 µl) were added to each well of a Nunc Lab-Tek 8-well chamber slide containing 200 µl (1∶5 dilution) (2.5×10^6^ CFU/ml) of DIS supplemented with 2, or 20 µg heme mL^−1^. The chamber slide was then incubated at 37°C with 5% CO_2_ and biofilms were grown for 48 or 72 hours with a media change every 24 hours. Following incubation, biofilms were prepared and visualized as previously described [31]. Biofilm structure and organization were imaged with an Axiovert 200M inverted epifluorescence microscope equipped with the Apotome attachment for improved fluorescence resolution and an Axiocam MRM CCD camera (Carl Zeiss Inc., Thornwood, NY). Three-dimensional renderings were performed in Image J [79] to generate orthogonal views and surface plots. Experiments were repeated in triplicate and representative samples of each replicate are shown.

For co-culture of heme-iron restricted and heme-iron replete 86-028NP, 86-028NP/pGM1.1 and 86-028NP/pKM1.1 strains were either continuously exposed or transiently restricted for heme-iron, mixed at either a 1∶1 or 1∶1000 ratio, and added (25 µl of each) to each well of a Nunc Lab-Tek 8-well chamber slide containing 200 µl of DIS supplemented with 2, or 20 µg heme mL^−1^ for a total inoculation of 2.5×10^6^ CFU/well. The chamberslide was incubated, washed and fixed and visualized as described [31], [33]. The biofilms were stained with live/dead DNA dye (Molecular Probes, Grand Island, NY) for the strains not containing a reporter plasmid. Experiments were repeated in triplicate and representative samples are shown.

### NTHI co-culture with chinchilla middle ear epithelial cells

Chinchilla middle ear epithelial (CMEE) cells were grown to confluence on a Nunc Lab-Tek 8-well chamber slide as previously described [33]. Epithelial cells were inoculated with 86-028NP/pGM1.1 bacteria that were either restricted or replete for heme-iron (10^6^ CFU in 200 µL DIS containing 2 µg heme mL^−1^ or CMEE media) [33], [80]. Seven hours after inoculation, the chambers were washed with DPBS and replaced with appropriate medium. The medium was replaced 24 hours after initial inoculation and cultured for an additional 24 hours. After 48 hours total culture, the cells were fixed in 4.0% paraformaldehyde (Electron Microscopy Sciences, Hatfield, PA) in DPBS (Mediatech, Manassas, VA) for fluorescence microscopy. For fluorescence microscopy, epithelial cell membranes were labeled with wheat germ agglutinin (WGA)-Alexafluor 594 (LifeTechnologies, Grand Island, NY). Biofilm structure, organization and NTHI localization were imaged with an Axiovert 200M inverted epifluorescence microscope equipped with the Apotome attachment for improved fluorescence resolution and an Axiocam MRM CCD camera (Carl Zeiss Inc., Thornwood, NY). Experiments were repeated in triplicate and representative samples are shown.

### Enrichment of filamentous NTHI

NTHI cultures, either continuously exposed or transiently restricted for heme-iron, were assessed for changes in cellular morphology as a result of heme-iron restriction by filter enrichment as previously described [36]. Briefly, the 86-028NP/pGM1.1 was either continuously exposed or transiently restricted for heme-iron for 24 h, and was added to each well of a Nunc Lab-Tek 8-well in DIS containing 2 µg heme mL^−1^ as described above to assess biofilm architecture after 48 hours of growth.

### Animal studies

#### Co-infection model

Healthy adult chinchillas (*Chinchilla lanigera*) purchased from Rauscher's Chinchilla ranch (LaRue, OH) with no evidence of middle ear infection were used to assess the biological consequence of heme-iron restriction on NTHI persistence. Chinchillas were anesthetized with xylazine (2 mg/kg, Fort Dodge Animal Health, Fort Dodge, IA) and ketamine (10 mg/kg) and middle ears challenged with NTHI GFP and mCherry reporter strains, either continuously exposed or transiently restricted for heme-iron, by transbullar inoculation. NTHI bacterial cultures (1∶10 mixture of one part 86-028NP/pKM1.1 (NTHI-mCherry; heme-iron restricted) and ten parts 86-028NP/pGM1.1 (NTHI-GFP; heme-replete) were obtained from overnight cultures, resuspended in 0.9% (w/v) sodium chloride in non-pyrogenic sterile water to an optical density of 0.65 measured at 490 nm, and diluted for inoculation. A total of 2130 microorganisms (1920 replete and 210 restricted) in 300 µl volume were inoculated into each ear. A total of 8 chinchillas were inoculated with each bacterial pair. Four animals were sacrificed 1 day post inoculation and the remaining four animals sacrificed 4 days post inoculation. Homogenates of 6 middle ear mucosa samples were plated for counts of viable microorganisms and analyzed for reporter strain activity to determine selectivity of infection from each timepoint. The remaining 2 middle ears were fixed in 4% paraformaldehyde in DPBS, decalcified overnight, paraffin embedded, sectioned and stained with hematoxylin and eosin for histologic examination at each timepoint.

#### Individual infection model

NTHI were obtained from overnight cultures, resuspended in 0.9% (w/v) sodium chloride in non-pyrogenic sterile water to an optical density of 0.65 measured at 490 nm, and diluted for inoculation. A total of 2100 replete or 270 restricted CFU of 86-028NP/pKM1.1 in 300 µl volume were inoculated into each ear. A total of 10 chinchillas were inoculated with either heme restricted or replete NTHI (5 chinchillas per cohort) and monitored daily by otoscopy and tympanometry. All animals were sacrificed 7 days post inoculation for a total of 6 middle ear mucosa tissue samples and 4 middle ears fixed for histologic examination for each cohort. Homogenates of middle ear mucosa were plated for counts of viable microorganisms to determine burden of infection. An additional two chinchillas were infected as described above and sacrificed at 4 days post infection for microscopic evaluation. Middle ear samples for histologic examination were fixed in 4% paraformaldehyde in DPBS, decalcified overnight, paraffin embedded, sectioned and stained with hematoxylin and eosin.

#### Competition infection model

To assess the biological impact of SulA function, chinchillas were co-infected with 450 CFU 86-028NP/pGZRS39A [78] and 450 CFU 86-028NPΔ*sulA*/pSPEC1 [76]. An equal mixture of bacteria was inoculated transbullarly into the middle ear of a chinchilla. Following 4, 7 and 10 days post-infection, middle ear effusions were collected by epitympanic taps through the superior bullae and directly obtained from the inferior bullae and monitored for survival by growth selective media. Homogenates of middle ear mucosa (day 12) were plated for counts of viable microorganisms to determine burden of infection.

#### RT-PCR analysis of *sulA* in NTHI

The transcription of *sulA* as part of an operon with *argR* was confirmed through the use of RT-PCR as previously described [29]. The primers used to amplify the junction between *argR* and *sulA*, 5′-GTGTTCCCAATACCAGTAGTCC-3′ and 5′-ATTCAACCAGTTGAGTAGTGAG. The primers to amplify *argR*, 5′-ACGTTGGATATTTTGCAATAAT-3′ and 5′-CTCGTGCTTTTAAAGAATTACT-3′. The primers used to amplify *sulA*, 5′- AGACCATGCCTGTTCGAATCAA-3′ and 5′-TTATTAACAGGAGGCACAGGGC-3′.

### Statistical analysis

Statistical analyses were performed using a two-tailed t-test or Mann-Whitney U-test as indicated (Graphpad Prism, LaJolla, CA).

## Supporting Information

Figure S1 **Tower architecture of transiently restricted cultures continues for 72 hours.** Biofilm growth was initiated as described in Figure 1 to provide examples from an additional experiment at 48 (**A, B**) and 72 (**C, D**) hours of growth. Biofilm base and tower height were measured following 72 hours of biofilm growth (**E**). Statistical analysis was performed using a paired t-test. High magnification image of transiently restricted 86-028NP to better depict the filamentous population within a 48 hour biofilm (**F**). Scale bar = 10 µm.
(TIF)

Figure S2 **Planktonic growth and adherence of continuously exposed and transiently restricted NTHI.** 86-028NP was grown in the presence (black square) or absence (gray circle) of heme-iron for 24 hours and subcultured into medium containing 2 µg heme mL^−1^. Growth was monitored every hour in a kinetic plate reader for 10 hours (**A**). Adherence of continuously exposed (CE) or transiently restricted (TR) NTHI to plastic surface was measured by crystal violet retention following 6 hours of growth in medium containing 2 µg heme mL^−1^ (**B**). 86-028NP (square) or 86-028NP *ΔsulA* (circle) were grown in the presence (black square) or absence (gray circle) of heme-iron for 24 hours and subcultured into medium containing 2 µg heme mL^−1^ (**C**).
(TIF)

Figure S3 **Schematic model that depicts the program of morphological and architectural changes that occur in response to transient heme iron restriction.** During heme-iron restriction we predict that a subpopulation of NTHI (green bacteria) initiate a programmatic change that manifests as changes in bacterial morphology upon restoration of heme-iron. In addition, the subpopulation produces the lace-like tower architecture observed (green bacteria) while the remainder of the population form the base of the biofilm (blue bacteria).
(TIF)

Figure S4 **SulA-related ortholog is not induced in the presence of DNA damage.** The magnitude of fluorescence emitted by GFP was measured following 5 hours of exposure to 0.5 or 2 µg/ml of Mitomycin C and normalized for cell number (OD_600_) (**A**). The magnitude of RNA expression of *argR* and *sulA* was determined by qRT-PCR following 30 or 60 minute exposure to 0.5 or 2 µg/ml mitomycin C (MMC) as described in the supplemental methods (**B**). Abbreviations: NTHI Promoter for P2 porin (P*_P2_*), promoterless *gfp* (P-), the promoter of the NTHI *argR-sulA* operon (P*_argRsulA_*), and the promoter for the UPEC SulA (P*_sulA_*).
(TIF)

Table S1 **Protein profiles of NTHI transiently restricted of heme-iron compared with NTHI continuously exposed of heme-iron.** Proteins that were identified by LC/MS/MS are indicated in the table with the common gene name and the NTHI gene number are included. The number indicates that number of peptides that were used to determine the protein identity.
(DOCX)

Methods S1 **Supplemental Methods for differential protein expression by 1D LC/MS/MS, reporter strain construction, assessment of promoter activity, RNA isolation and RT-PCR analysis.** This text includes the detailed methodologies for all of the data included in the supplemental materials.
(DOC)
