# Supplementary figures and images for: Haemophilus Responses to Nutritional Immunity: Epigenetic and Morphological Contribution to Biofilm Architecture, Invasion, Persistence and Disease Severity
Source: PLoS Pathog. 2013 Oct 10;9(10):e1003709. doi: 10.1371/journal.ppat.1003709 (PMC3795038; doi:10.1371/journal.ppat.1003709)

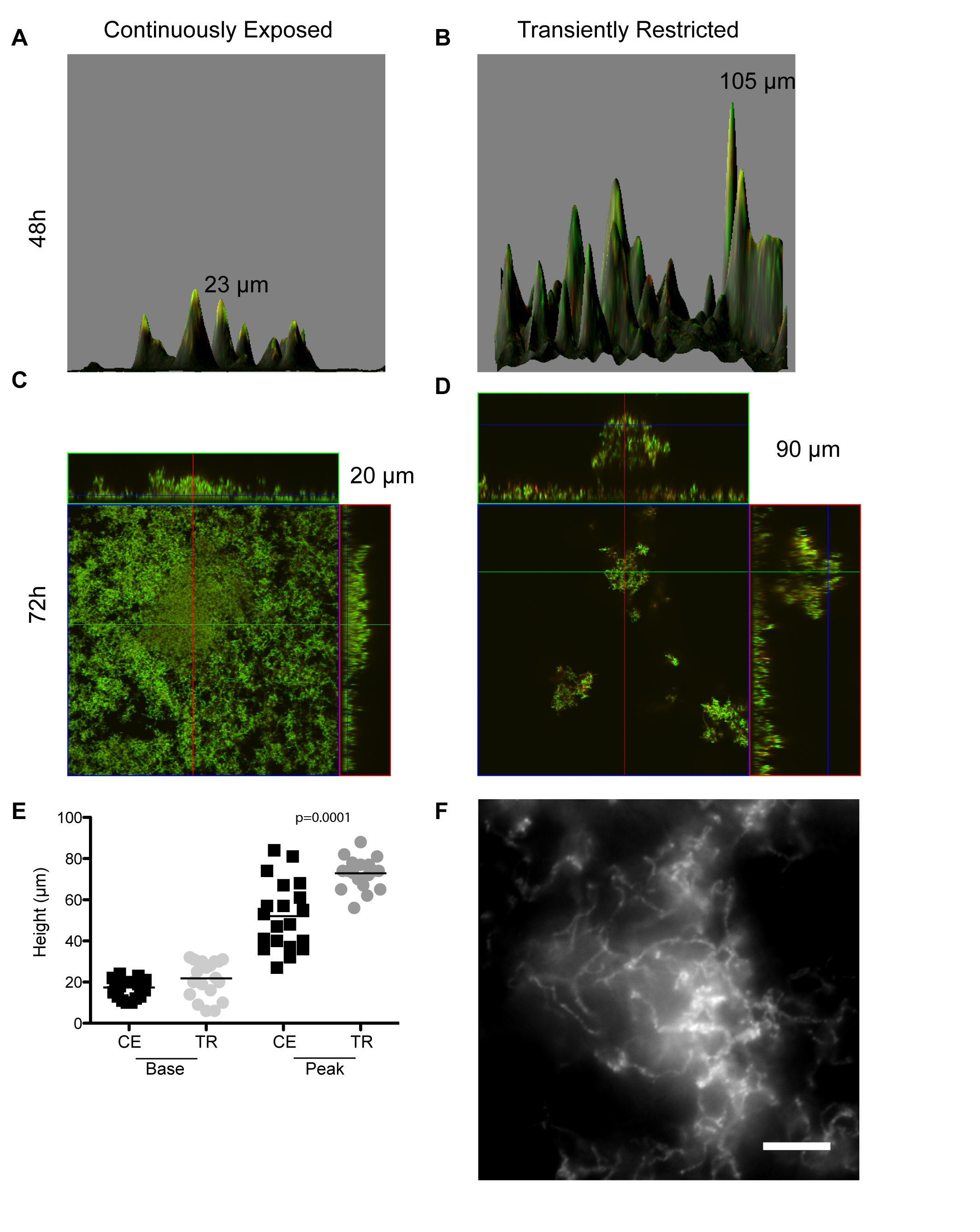

Supplement: Figure S1 — Tower architecture of transiently restricted cultures continues for 72 hours. Biofilm growth was initiated as described in Figure 1 to provide examples from an additional experiment at 48 (A, B) and 72 (C, D) hours of growth. Biofilm base and tower height were measured following 72 hours of biofilm growth (E). Statistical analysis was performed using a paired t-test. High magnification image of transiently restricted 86-028NP to better depict the filamentous population within a 48 hour biofilm (F). Scale bar = 10 µm. (TIF) [file ppat.1003709.s001.tif]

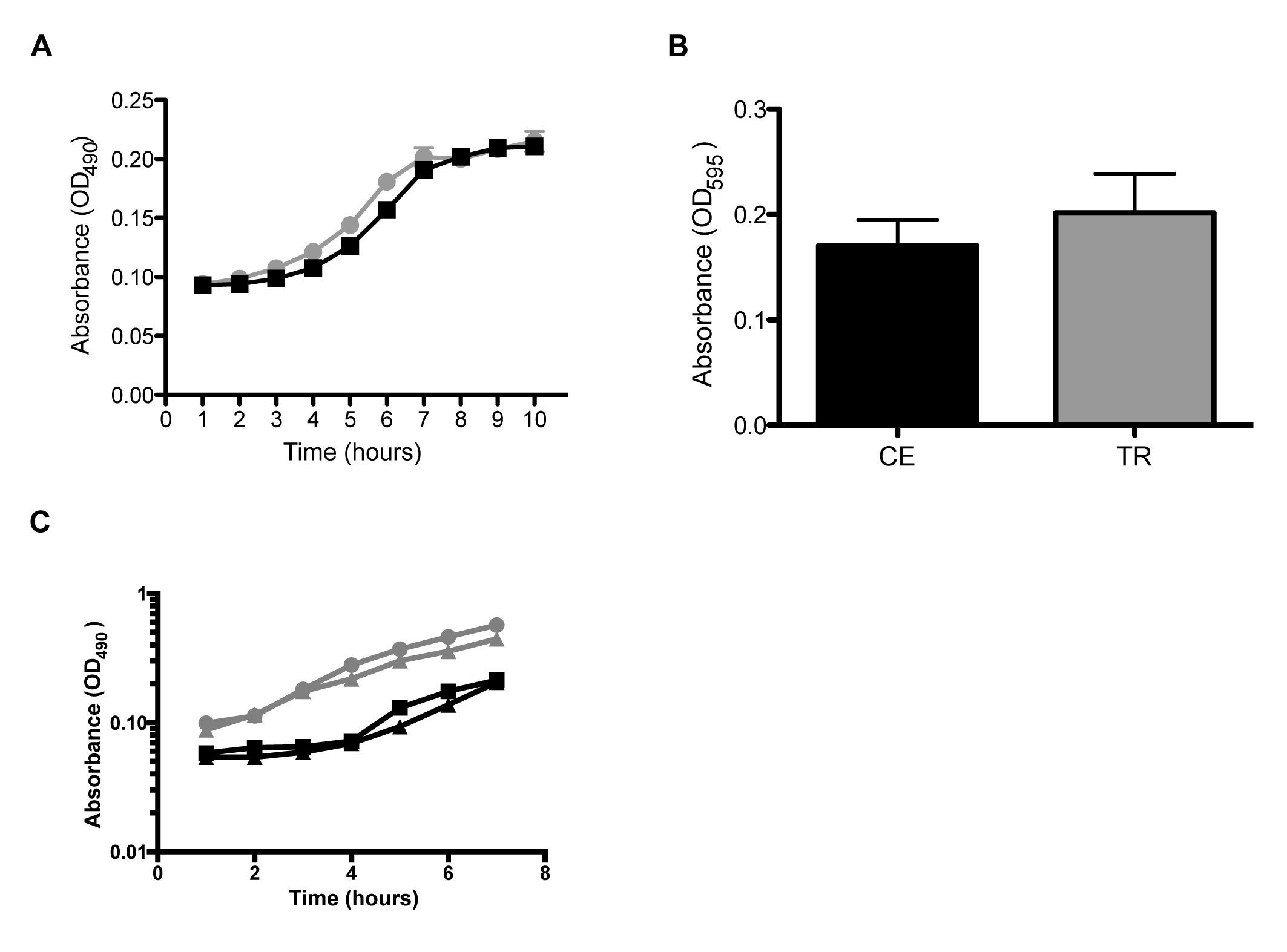

Supplement: Figure S2 — Planktonic growth and adherence of continuously exposed and transiently restricted NTHI. 86-028NP was grown in the presence (black square) or absence (gray circle) of heme-iron for 24 hours and subcultured into medium containing 2 µg heme mL−1. Growth was monitored every hour in a kinetic plate reader for 10 hours (A). Adherence of continuously exposed (CE) or transiently restricted (TR) NTHI to plastic surface was measured by crystal violet retention following 6 hours of growth in medium containing 2 µg heme mL−1 (B). 86-028NP (square) or 86-028NP ΔsulA (circle) were grown in the presence (black square) or absence (gray circle) of heme-iron for 24 hours and subcultured into medium containing 2 µg heme mL−1 (C). (TIF) [file ppat.1003709.s002.tif]

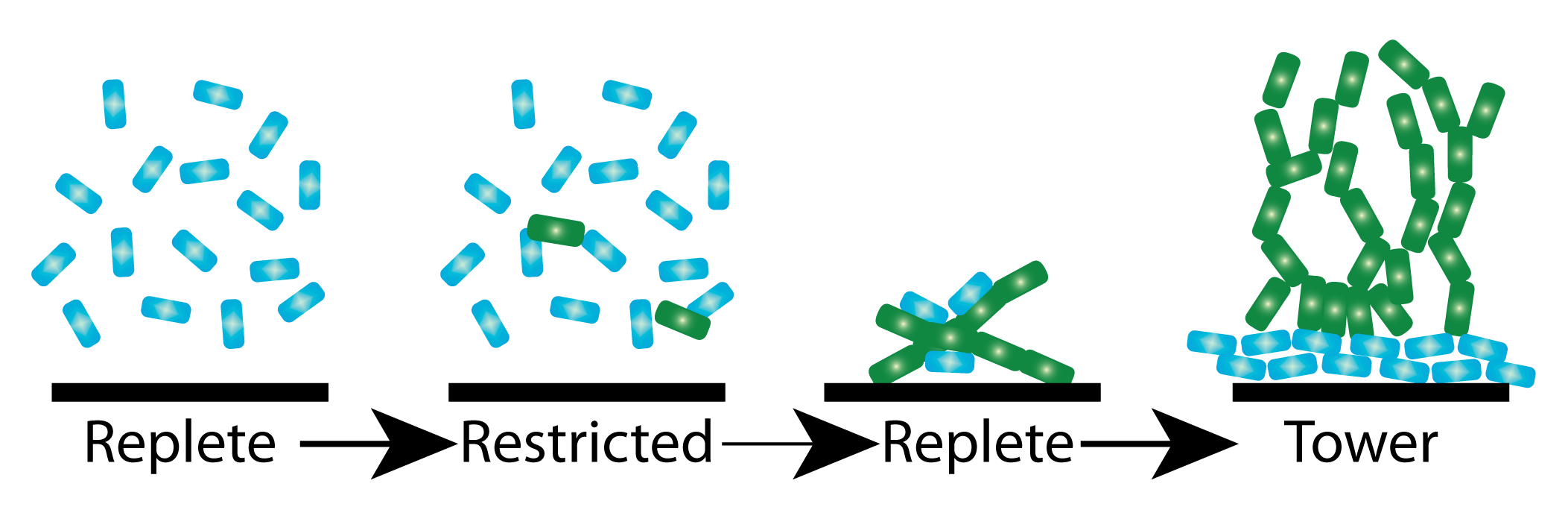

Supplement: Figure S3 — Schematic model that depicts the program of morphological and architectural changes that occur in response to transient heme iron restriction. During heme-iron restriction we predict that a subpopulation of NTHI (green bacteria) initiate a programmatic change that manifests as changes in bacterial morphology upon restoration of heme-iron. In addition, the subpopulation produces the lace-like tower architecture observed (green bacteria) while the remainder of the population form the base of the biofilm (blue bacteria). (TIF) [file ppat.1003709.s003.tif]

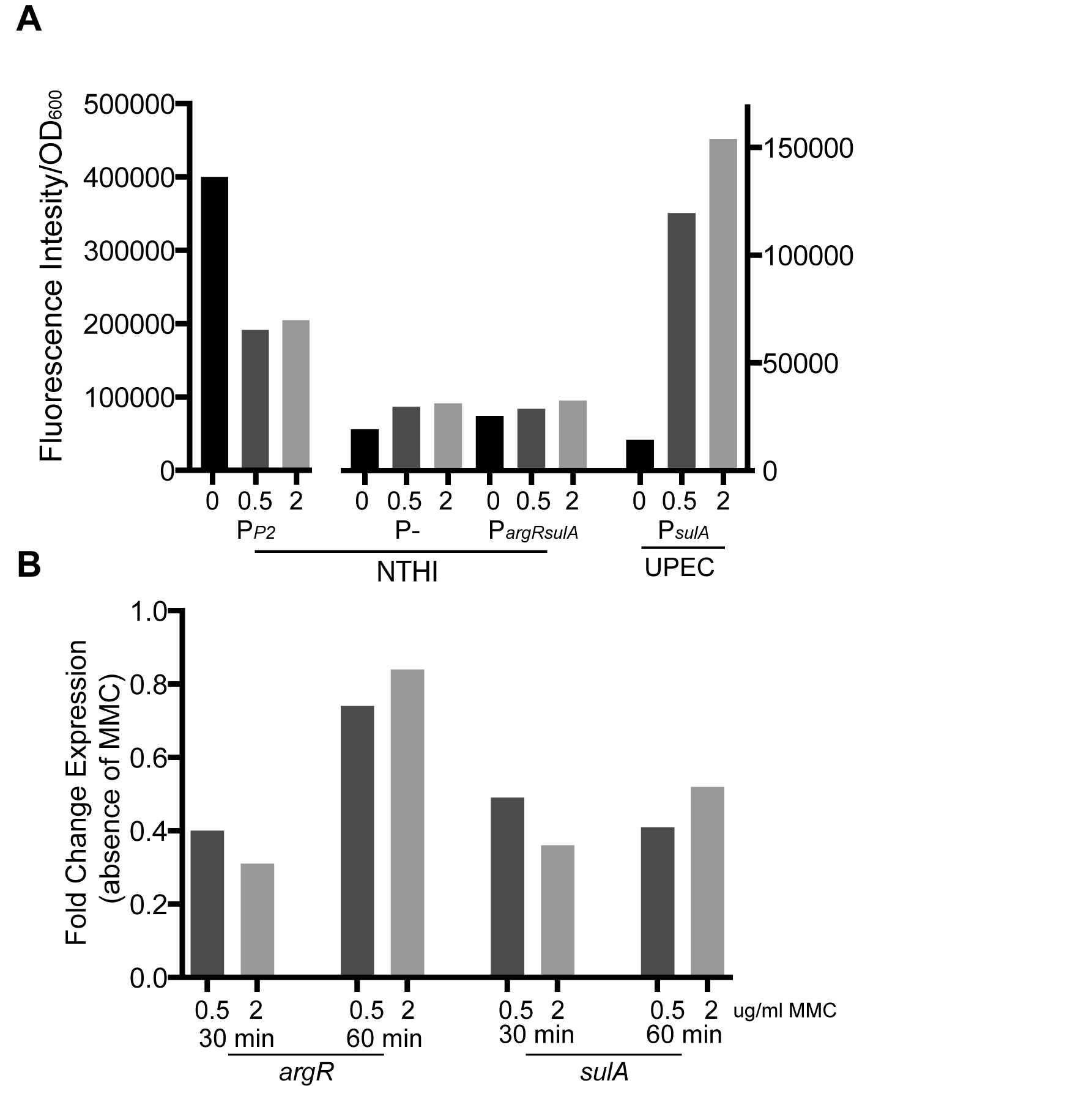

Supplement: Figure S4 — SulA-related ortholog is not induced in the presence of DNA damage. The magnitude of fluorescence emitted by GFP was measured following 5 hours of exposure to 0.5 or 2 µg/ml of Mitomycin C and normalized for cell number (OD600) (A). The magnitude of RNA expression of argR and sulA was determined by qRT-PCR following 30 or 60 minute exposure to 0.5 or 2 µg/ml mitomycin C (MMC) as described in the supplemental methods (B). Abbreviations: NTHI Promoter for P2 porin (PP2), promoterless gfp (P-), the promoter of the NTHI argR-sulA operon (PargRsulA), and the promoter for the UPEC SulA (PsulA). (TIF) [file ppat.1003709.s004.tif]
