# Supplementary material for: Haemophilus Responses to Nutritional Immunity: Epigenetic and Morphological Contribution to Biofilm Architecture, Invasion, Persistence and Disease Severity
Source: PLoS Pathog. 2013 Oct 10;9(10):e1003709. doi: 10.1371/journal.ppat.1003709 (PMC3795038; doi:10.1371/journal.ppat.1003709)
Supplement: Table S1 — Protein profiles of NTHI transiently restricted of heme-iron compared with NTHI continuously exposed of heme-iron. Proteins that were identified by LC/MS/MS are indicated in the table with the common gene name and the NTHI gene number are included. The number indicates that number of peptides that were used to determine the protein identity. (DOCX) [file ppat.1003709.s005.docx]

| Accession Number | NTHI gene # | Gene name | Protein function | Peptide count | ANOVA | T-test |
| --- | --- | --- | --- | --- | --- | --- |
| YP_248373.1 | NTHI0804 | *ilvC* | ketol-acid reductoisomerase | 10 | 0.000 | 0.003 |
| YP_248698.1 | NTHI1178 | *trmE, mnmE* | tRNA modification GTPase | 1 | 0.006 | 0.000 |
| YP_247874.1 | NTHI0243 | *acpP* | acyl carrier protein | 3 | 0.009 | 0.044 |
| YP_248393.1 | NTHI0831 | *tnaA* | tryptophanase | 2 | 0.012 | 0.051 |
| YP_248611.2 | NTHI1080 | *rpsB* | 30S ribosomal protein S2 | 18 | 0.017 | 0.025 |
| YP_248753.1 | NTHI1247 |  | ABC transporter | 1 | 0.021 | 0.002 |
| YP_248559.1 | NTHI1021 | *hbpA* | Glutathione binding protein | 14 | 0.026 | 0.020 |
| YP_248111.1 | NTHI0517 |  | hypothetical protein NTHI0517 | 1 | 0.026 | 0.053 |
| YP_248588.1 | NTHI1054 | *glyA* | serine hydroxymethyltransferase | 7 | 0.029 | 0.062 |
| YP_248510.1 | NTHI0965 | *rpoA* | DNA-directed RNA polymerase subunit alpha | 12 | 0.029 | 0.071 |
| YP_248801.1 | NTHI1308 | *ddl* | D-alanine--D-alanine ligase | 2 | 0.030 | 0.029 |
| YP_248515.1 | NTHI0972 | *frr* | ribosome recycling factor | 2 | 0.051 | 0.039 |
| YP_248507.1 | NTHI0962 | *rpsM* | 30S ribosomal protein S13 | 7 | 0.078 | 0.032 |
| YP_248827.1 | NTHI1335 | *serC* | phosphoserine aminotransferase | 2 | 0.087 | 0.009 |
| YP_248262.1 | NTHI0681 | *gnd* | 6-phosphogluconate dehydrogenase | 7 | 0.087 | 0.051 |
| YP_247907.1 | NTHI0284 | *fur* | ferric uptake regulation protein | 1 | 0.087 | 0.299 |

Table S1. Proteins that were determined to be statistically significantly produced at different levels following transient restriction of heme-iron are listed. The number of peptides sequenced from each protein are indicated as well as the statistical analyses.
