# Supplementary material for: Haemophilus Responses to Nutritional Immunity: Epigenetic and Morphological Contribution to Biofilm Architecture, Invasion, Persistence and Disease Severity
Source: PLoS Pathog. 2013 Oct 10;9(10):e1003709. doi: 10.1371/journal.ppat.1003709 (PMC3795038; doi:10.1371/journal.ppat.1003709)
Supplement: Methods S1 — Supplemental Methods for differential protein expression by 1D LC/MS/MS, reporter strain construction, assessment of promoter activity, RNA isolation and RT-PCR analysis. This text includes the detailed methodologies for all of the data included in the supplemental materials. (DOC) [file ppat.1003709.s006.doc]

Supplemental Methods

**Differential Protein Expression by 1D LC/MS/MS**

Six biological samples, n=3 each of “TR” and “CE” conditions, were lysed in 50 mM ammonium bicarbonate (AmBic) using a high pressure cell (Constant Systems LTD, Kennesaw, GA). Samples were supplemented to a final concentration of 0.1% w/v Rapigest surfactant (Waters Corporation) and approximately 50 ug of each sample was reduced, alkylated and digested with trypsin according to our standardized protocol . After digestion, all samples were spiked with ADH1_YEAST digest (Massprep standard, Waters Corporation) as a surrogate standard (50 fmol ADH per ug total protein), dried and resuspended in 2% acetonitrile, 0.1% TFA (pH 2.5) at a concentration of 0.5 ug/uL digested protein. Quantitative LC/MS/MS was performed on 1 μg of protein digest per sample, using a nanoAcquity UPLC system (Waters Corp) coupled to a Synapt G2 HDMS high resolution accurate mass tandem mass spectrometer (Waters Corp.) via a nanoelectrospray ionization source. Briefly, the sample was first trapped on a Symmetry C18 300 um × 180 mm trapping column (5 μl/min at 99.9/0.1 v/v water/acetonitrile), after which the analytical separation was performed using a 1.7 um Acquity BEH130 C18 75 um × 250 mm column (Waters Corp.) using a 90-min gradient of 5 to 40% acetonitrile with 0.1% formic acid at a flow rate of 300 nanoliters/minute (nL/min) with a column temperature of 45C. Data collection on the Synapt G2 mass spectrometer was performed in ion-mobility assisted data-independent acquisition (HDMSE) mode, using 0.6 second alternating cycle time between low (6V) and high (27-50V) collision energy (CE). Scans performed at low CE measure peptide accurate mass and intensity (abundance), while scans at elevated CE allow for qualitative identification of the resulting peptide fragments via database searching.

The QC pool containing equivalent amounts of all 6 samples was used to condition the UPLC column prior to the study and was run 3 times throughout the study to obtain QC reproducibility metrics (for a total of 9 quantitative analyses). Following the LC/MS/MS analyses, data was imported into Rosetta Elucidator v3.3 (Rosetta Biosoftware, Inc), and all LC/MS runs were aligned based on the accurate mass and retention time of detected ions (“features”) using PeakTeller algorithm (Elucidator). The relative peptide abundance was calculated based on area-under-the-curve (AUC) of aligned features across all runs. The overall dataset had 71,358 quantified features, and high collision energy (peptide fragment) data was collected in 57,549 spectra for sequencing by database searching by either IdentityE search engine (HDMSE, 55,241 spectra) or Mascot v2.2 (DDA, 2,308 spectra). This MS/MS data was searched against a the NCBI RefSeq database for *Haemophilus Influenzae strain 86-028 NP* (1726 protein sequences, downloaded http://www.ncbi.nlm.nih.gov/pubmed on Sept 6, 2012). This database also contained a reversed-sequence “decoy” database for false positive rate determination. Data was searched with 10 ppm precursor and 0.04 Da product ion mass accuracy, fixed modification of Cys (carbamidomethylation), and variable modifications including oxidation (Met) and deamidation (Asn, Gln). After individual peptide scoring using PeptideProphet algorithm (Elucidator), the data was annotated at a <1% peptide false discovery rate. This analysis yielded identifications for 2613 peptides and 422 proteins across all samples, including 300 proteins with two or more peptides quantified. For quantitative processing, the data was first curated to contain only high quality peptides with appropriate chromatographic peak shape (peak time score >0.75, peak m/z score >0.8, peak width >0.1) and the dataset was intensity scaled to the robust mean across all samples analyzed; the final quantitative dataset was based on 2488 peptides and contains 417 proteins.

**Reporter Strain Construction and Assessment of Promoter Activity**

The influence of mitomycin C (MMC) exposure on NTHI *argR-sulA* promoter activity and *E. coli* *sulA* promoter activity was determined by monitoring GFP production from NTHI and *E. coli* reporter strains in response to bacterial exposure to MMC. The *E. coli* *sulA-gfp* reporter fusion was constructed as previously described . Briefly, a 373 base pair fragment containing the LexA box of *sulA* was inserted immediately upstream of a promoterless *gfp*. A single copy of the reporter was inserted into the inactive *attB* site. The NTHI *argR-sulA-gfp* reporter fusion was constructed by cloning the *argR-sulA* promoter region upstream *gfpmut3* in plasmid pRSM2169, constructed as previously described . Briefly, primers 5’-GCGCGGATCCAACAAGTGCTTTGCCGATAAG-3’ and 5’-GCGCGCATGCAAATACTCCTTATGTGTTAGT-3’ were used to amplify the promoter region upstream *argR* which extended 51 bases into the 5’ end of *sulA*. The primers contained a *Sph*I and *Bam*HI site that were digested and ligated into *Sph*I and *Bam*HI digested pRSM2169. The ligation was transformed into NTHI and transformants were screened for the correct plasmid and saved as pDRH001. Plasmid construct was confirmed by DNA sequencing.

NTHI strains were plated on chocolate agar II overnight at 37°C in 5% CO_2_. NTHI was adjusted to an OD_490_ of 0.65 and diluted 1:1000 in prewarmed brain heart infusion broth supplemented with 2 µg heme/ml (Becton, Dickinson, Sparks, MD). After 3 hours of growth, MMC was added to a final concentration of 0.5 or 2 µg/ml for an additional 5 hours of growth. UTI89 *attB*::P*_sulA_* –*gfp* was growth to saturation in Luria Broth (Fisher Scientific) at 37°C. The UPEC strain was then diluted 1:500 in fresh Luria Broth. After 1.5 hours of growth, MMC was added to a final concentration of 0.5 or 2 µg/ml for an additional 5 hours of growth. Cultures were concentrated by centrifugation at 9,000 x g for 5 minutes and resuspended in one tenth volume. The absorbance (OD600) and fluorescence emission (excitation 485 nm, emission 528 nm) were analyzed using a Synergy H1 hybrid reader (BioTek, Winook, VT). Other groups have used 0.1 to 8 µg/ml of MMC or other DNA damaging agents (e.g. UV light) to determine genes that are induced in other bacterial species as part of the SOS response (including *sulA*) . These conditions typically result in loss of up to 2 logs in viability.

**RNA Isolation and RT-PCR**

NTHI strain 86-028NP was grown overnight on Chocolate II agar (Becton, Dickinson, Sparks, MD) or LB broth and then subcultured into prewarmed brain heart infusion broth supplemented with 2 µg heme/ml (Becton, Dickinson, Sparks, MD) and 1 µg NAD/ml (Becton, Dickinson, Sparks, MD) or LB broth, respectively. Cultures were grown static for 3 h at 37°C to logarithmic phase and exposed to 0.5 µg/ml or 2 µg/ml mitomycin C and subsequently incubated at 37°C for 30 and 60 minutes. Bacterial cell cultures were pelleted at 4°C for 10 min at 3,220 × *g* and RNA purified using TRIzol reagent (Life Technologies, Grand Island, NY) as outlined in Mason *et al*. . The RNA concentration was determined using a NanoDrop spectrophotometer (NanoDrop Products, Wilmington, DE), and each sample was analyzed for integrity using an Agilent 2100 bioanalyzer (Agilent Technologies, Santa Clara, CA) to confirm the absence of degraded RNA.  qRT-PCR was performed with a one-step QuantiTect SYBR green RT-PCR kit (Qiagen, Valencia, CA). Two biological replicates and two technical replicates were performed for each gene analyzed. Number of RNA copies for each gene were calculated in 1ng RNA, compared to a standard genomic DNA curve generated for each primer pair.

1. Ralston-Hooper KJ*, et al.* (2012) Application of a Label-free, Gel-free Quantitative Proteomics Method for Ecotoxicological Studies of Small Fish Species. *Environmental science & technology*.

2. Justice SS, Hunstad DA, Seed PC, & Hultgren SJ (2006) Filamentation by Escherichia coli subverts innate defenses during urinary tract infection. *Proc Natl Acad Sci U S A* 103(52):19884-19889.

3. Mason KM, Munson RS, Jr., & Bakaletz LO (2003) Nontypeable *Haemophilus influenzae* gene expression induced *in vivo* in a chinchilla model of otitis media. *Infect Immun* 71(6):3454-3462.

4. Quillardet P, Rouffaud MA, & Bouige P (2003) DNA array analysis of gene expression in response to UV irradiation in Escherichia coli. *Research in microbiology* 154(8):559-572.

5. Jin H, Retallack DM, Stelman SJ, Hershberger CD, & Ramseier T (2007) Characterization of the SOS response of Pseudomonas fluorescens strain DC206 using whole-genome transcript analysis. *FEMS microbiology letters* 269(2):256-264.

6. Biran A*, et al.* (2011) Microbial genotoxicity bioreporters based on sulA activation. *Analytical and bioanalytical chemistry* 400(9):3013-3024.

7. Ogino H, Teramoto H, Inui M, & Yukawa H (2008) DivS, a novel SOS-inducible cell-division suppressor in Corynebacterium glutamicum. *Mol Microbiol* 67(3):597-608.

8. Ulrich RL*, et al.* (2013) Characterization of the Burkholderia thailandensis SOS response using whole transcriptome shotgun sequencing. *Appl Environ Microbiol*.

9. Mason KM, Bruggeman ME, Munson RS, & Bakaletz LO (2006) The non-typeable Haemophilus influenzae Sap transporter provides a mechanism of antimicrobial peptide resistance and SapD-dependent potassium acquisition. *Mol Microbiol* 62(5):1357-1372.
